# Sensitive Determination of Proteolytic Proteoforms in Limited Microscale Proteome Samples*[Fn FN2]

**DOI:** 10.1074/mcp.TIR119.001560

**Published:** 2019-08-30

**Authors:** Samuel S. H. Weng, Fatih Demir, Enes K. Ergin, Sabrina Dirnberger, Anuli Uzozie, Domenic Tuscher, Lorenz Nierves, Janice Tsui, Pitter F. Huesgen, Philipp F. Lange

**Affiliations:** ‡ Department of Pathology and Laboratory Medicine, University of British Columbia, Vancouver, Canada; § Michael Cuccione Childhood Cancer Research Program, BC Children's Hospital, Vancouver, Canada; ¶ Central Institute for Engineering, Electronics and Analytics, ZEA-3, Forschungszentrum Jülich, Germany; ‖ Cologne Excellence Cluster on Cellular Stress Responses in Aging Associated Diseases, Medical Faculty and University Hospital, University of Cologne, Cologne, Germany

**Keywords:** N-terminal modifications, proteases, post-translational modifications, substrate identification, cell sorting, clinical proteomics, proteolysis, subcellular analysis, enrichment, N termini

## Abstract

Protein N termini unambiguously identify truncated, alternatively translated or modified proteoforms with distinct functions and reveal perturbations in disease. Selective enrichment of N-terminal peptides is necessary to achieve proteome-wide coverage for unbiased identification of site-specific regulatory proteolytic processing and protease substrates. However, many proteolytic processes are strictly confined in time and space and therefore can only be analyzed in minute samples that provide insufficient starting material for current enrichment protocols. Here we present High-efficiency Undecanal-based N Termini EnRichment (HUNTER), a robust, sensitive and scalable method for the analysis of previously inaccessible microscale samples. HUNTER achieved identification of >1000 N termini from as little as 2 μg raw HeLa cell lysate. Broad applicability is demonstrated by the first N-terminome analysis of sorted human primary immune cells and enriched mitochondrial fractions from pediatric cancer patients, as well as protease substrate identification from individual *Arabidopsis thaliana* wild type and Vacuolar Processing Enzyme-deficient mutant seedlings. We further implemented the workflow on a liquid handling system and demonstrate the feasibility of clinical degradomics by automated processing of liquid biopsies from pediatric cancer patients.

## Abstract (precis)

Protein N termini reveal fundamental regulatory mechanisms and their perturbation in disease. Regulatory proteolysis is often spatially and temporally confined, thus accessible only in minimal specimen incompatible with established protocols. We developed a robust, sensitive, scalable and automatable method for system-wide identification of thousands of N termini from minute samples. Applications revealed distinct N-terminal profiles in sorted immune cells and mitochondria from pediatric cancer patient cells, protease substrates in *Arabidopsis* seedlings and effects of chemotherapy on proteolytic proteoforms in clinical liquid biopsies.

## Graphical Abstract

**Figure:**
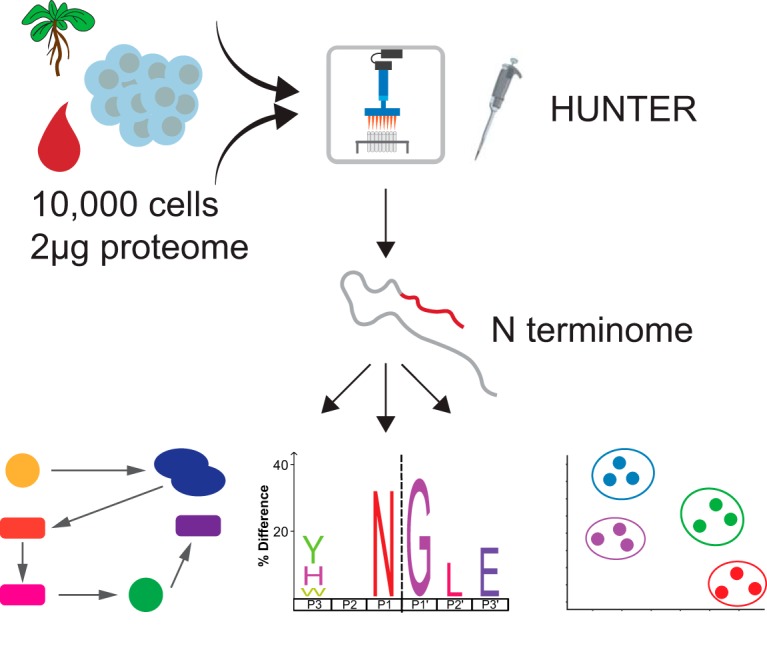

**Highlights**

- Single-pot workflow for manual or automated enrichment of N-terminal peptides.
- Sensitive enrichment of protein N termini from 10,000 cells or 2 μg crude proteome.
- Data independent acquisition improves precision of peptide level quantification.
- First degradomic analyses of sorted immune cells, single seedlings, and mitochondria from patient cells.

Protein N termini define different proteoforms arising from limited proteolytic processing, alternative translation initiation and co- or post-translational N-terminal modification (1). De-regulated proteolytic processing of proteins is a well-known driver of disease resulting in aberrant activation, inactivation or change in function, stability or localization of the protein (2). Consequently, proteases are considered promising drug targets (3, 4) and proteolytic proteoforms may be used as clinical biomarkers (5). Current peptide-based bottom-up proteomics enables protein identification and quantification on a proteome-wide scale (6), but standard protocols and database search parameters exclude N termini identification, particularly of the protease-generated neo-N-terminal peptides (7). To overcome this challenge, dedicated methods for selective enrichment and unbiased identification of N-terminal peptides from complex proteomes have been developed (8–12). Such N-terminome profiling has greatly advanced our understanding of apoptosis (10, 13), revealed novel proteolytic proteoforms in human tissues (14, 15) and animal models of disease (16), identified protease substrates underlying common disease and rare genetic disorders (17, 18) and enabled characterization of alternative protein translation initiation sites (19, 20) and protein N-terminal modifications (21). However, critical proteolytic processes in development (22) and disease pathogenesis (23) are strictly confined in space and time. With current N termini enrichment technologies, such processes cannot be characterized because of insufficient starting material (24). The most sensitive protocol available to date enabled N termini enrichment from 40 μg of 10 pooled isobarically labeled, purified proteomes obtained from milligrams of cultured cell lysate (25). In contrast, improved and automated proteome (26) and phosphoproteome (27) sample processing now enables comprehensive analyses of cell-type specific processes and clinically relevant microscale samples (<20 μg). To achieve a similar leap in the analysis of proteolytic proteoforms, we have developed High-efficiency Undecanal-based N Termini EnRichment (HUNTER)^1^, an automatable workflow for the sensitive enrichment of N-terminal peptides from as little as 2 μg crude protein in any cell or tissue lysate using off-the-shelf reagents (Fig. 1*A*).

## EXPERIMENTAL PROCEDURES

### 

#### 

##### Experimental Design and Statistical Rationale

Sample type and size and as well as data acquisition, processing and statistical analysis are detailed and justified for the individual experiments in the following sections. All data is publicly available and respective repositories and accession numbers are listed at the end.

##### Cell Culture and Human Samples

HeLa cells (American Type Culture Collection; cat. no. CCL-2) were cultured in RPMI 1640 medium (ThermoFisher Scientific; cat. no. 11875–093) with 10% Cosmic Calf Serum (GE Healthcare Life Sciences; cat. no. SH30087.04) and maintained in a humidified incubator at 37 °C with 5% CO_2_. Cultured cells were collected using 0.25% Trypsin-EDTA (ThermoFisher Scientific; cat. no. 25200056), centrifuged at 800 × *g* and washed with PBS (ThermoFisher Scientific; cat. no. 10010023) to collect pellets of different cell quantities. Cell pellets were frozen and stored in −80 °C freezer until further lysis. B-cell acute lymphoblastic leukemia (B-ALL) cell lines 380 (ACC 39) and 697 (ACC 42) cells were procured from DSMZ (Braunschweig, Germany). B-ALL cell lines were cultured in RPMI 1640 media supplemented with 10% heat-inactivated fetal bovine serum (ThermoFisher Scientific; cat. no. 10082147) and 2 mm l-Glutamine (ThermoFisher Scientific; cat. no. 25030081) and maintained at 37 °C in 5% CO_2_. Commercial human blood plasma was purchased from STEMCELL Technologies (cat. no. 70039). Primary pediatric B-ALL and AML patient mononuclear cells enriched from bone marrow aspirates, plasma (BP) and bone marrow interstitial fluid (BM) were retrospectively sourced from the Biobank at BC Children's Hospital (BCCH) following informed consent and approval by the University of British Columbia Children's and Women's Research Ethics Board (REB #H15-01994) in agreement with the Declaration of Helsinki. Patient BP and BM samples were collected at the time of diagnosis (D0) and 29 days after induction chemotherapy (D29). Peripheral blood mononuclear cells (PBMC) from healthy donors were obtained following informed consent and approval by the University of British Columbia Children's and Women's Research Ethics Board (REB #H10-01954). Individual populations were obtained by Fluorescence Activated Cell Sorting using the following antibody combinations: CD19+ for B-cells, CD14+ for monocytes, CD3- CD56+ for natural killer (NK) cells and CD3+ CD56+ for NK T-cells (NKT cells).

##### Plant Material

*Arabidopsis thaliana* Col-8 wild type (accession N60000) and VPE0 mutant (accession N67918) seed stocks were obtained from the Nottingham Arabidopsis Stock Center (NASC, Nottingham, UK). *A. thaliana* Col-8 plants were stratified for 3d at 4 °C and subsequently grown on soil at short day conditions (9 h light with an intensity of 100 μE m^−2^ s^−1^ at 22 °C and 15 h darkness at 18 °C, 75% RH). Leaves of 6-week-old plants were harvested and snap frozen in liquid nitrogen. For seedling experiments, *A.thaliana* seeds were stratified for 3 d at 4 °C and germinated for 2.5 d (5 d for single seedling experiment) on filter paper at a short day time regime (9 h light with an intensity of 110 μE m^−2^ s^−1^ at 22 °C and 15 h darkness at 18 °C).

##### Rat Brain Samples

Rat brains were obtained from Wistar rats that were sacrificed for liver perfusion experiments at the University Hospital Düsseldorf as approved by local authorities (LANUV NRW #G287/15) and immediately snap frozen in liquid nitrogen.

##### Preparation of Stage-tips

Four small circular Empore™ SPE C18 disks (Sigma, cat. no. 66883-U) were punched with a flat-end needle (Hamilton, cat. no. 90517). A straightened paper clip was used to gently push down the C18 disks into a P200 pipette tip (VWR, cat. no. 89079-474).

##### High-pH Reversed Phase Fractionation

Fractionation was performed with an Agilent 1100 HPLC system equipped with a diode array detector (254, 260, and 280 nm). HPLC system was installed with a Kinetic EVO C18 column (2.1 mm×150 mm, 1.7 μm core shell, 100Å pore size, Phenomenex). The samples were run at a flow rate of 0.2 ml per minute using a gradient of mobile phase A (10 mm ammonium bicarbonate, pH 8, Fisher Scientific, cat. no. BP2413-500) and mobile phase B (acetonitrile, Sigma-Aldrich, cat. no. 34998-4L) from 3% to 35% B over 60 min. Fractions were collected every minute across the elution window for a total of 48 fractions, then concatenated to a final set of 12 (*e.g.* fraction 1 + 13 + 25 + 37 as final fraction 1). All the fractions were dried in a SpeedVac centrifuge and resuspended in 0.1% FA in water (Thermo Scientific, cat. no. SC2352911) prior to mass spectrometry analysis.

Terminal amine isotopic labeling of substrates was performed according to the detailed terminal amine isotopic labeling of substrates (TAILS) procedure as described in the official TAILS bench protocol v4 by the Overall Lab (http://clip.ubc.ca/resources/protocols-and-sops/, May 2016). Briefly, HeLa cells were first lysed with 6 m guanidine hydrochloride (GuHCl, Fisher BioReagents, cat. no. BP178–1) to a final concentration of 3 m in a 1.5 ml protein Lobind tube (Eppendorf, cat. no. 022431081). Then, DTT (Fisher BioReagents, cat. no. BP172-25) and IAA (Sigma, cat. no. I6125-25G) were introduced in the reduction and alkylation steps. Dimethyl labeling was used to block amine groups within the proteome. Subsequently, trypsin (1 mg/ml, Promega, cat. no. V5113) was introduced and incubated at 37 °C overnight. Amine-reactive hyperbranched aldehyde-derivatized polymer (HPG-ALD polymer, https://www.flintbox.com/public/project/1948/) was used to tag newly formed internal tryptic peptides. Finally, the tagged-internal peptides were isolated by stage-tips. Protein precipitation was performed between each labeling step.

### High-efficiency Undecanal based N Termini EnRichment (HUNTER)

#### 

##### Preparation of HeLa and Peripheral Blood Mononuclear Sorted Cell Lysates

The HeLa cell and sorted cell samples were first lysed in a 1.5 ml protein Lobind tube lysis buffer consisting of 1% sodium dodecyl sulfate (Fisher BioReagents, cat. no. BP8200-500) and 2× Thermo Halt protease inhibitor mixture (Thermo Scientific, cat. no. 1861279) in 50 mm HEPES, pH 8.0 (Sigma, cat. no. H4034-1KG). The lysate was heated at 95 °C for 5 min, then chilled on ice for another 5 min. Any liquid condensation or droplets was spun down by centrifugation. Benzonase (EMD Millipore, cat. no. 70664-3) was added at a ratio of 1 unit to 37 μg of DNA and incubated at 37 °C for 30 min. Then DTT was added to 10 mm and incubated at 37 °C for 30 min, followed by addition of 2-chloroacetamide (CAA; Sigma-Aldrich, cat. no. C0267-100G) to 50 mm and further incubation at RT in the dark for 30 min. To quench the alkylation, DTT was added to a final concentration of 50 mm and incubate at RT in the dark for 20 min. Protein Lobind tubes were used during all sample handling steps.

##### Preparation of Mitochondrial Enrichment Samples

Mitochondrial enrichment was performed on 2.5 million cells from two B-cell lines (697, 380), and 2.5 million bone marrow monocytes from a pediatric AML patient (AML-1). All samples were processed in technical replicates (*n* = 2 or *n* = 3). Cells in mitochondrial isolation buffer (1 mm EGTA/HEPES pH 7.4, 200 mm Sucrose, 1× Halt protease inhibitor) were disrupted by Pressure Cycling Technology (PCT) using a Barocycler EXT2320 and a PCT 30 μl MicroTube (Pressure BioSciences, Easton, Massachusetts, United States). The cell samples were homogenized and lysed using 15 cycles of 25kpsi for 20 s and followed by 20 s at ambient pressure at 26 °C. Cells were subsequently centrifuged at 900 × *g* and the pellet fraction (Mitochondrial fraction 1, M1) was collected. The supernatant was transferred to a new tube and centrifuged at 13,000 × *g* to collect the second pellet fraction (Mitochondrial fraction 2, M2) and cytosolic supernatant (cytosolic fraction, C). Pellet fractions M1 and M2 made up the mitochondrial enriched portion. Proteins were reduced and denatured as described for HeLa samples.

##### Preparation of Arabidopsis thaliana Seedling Lysates

Single 5 day-old *Arabidopsis* seedlings, or three 2.5 day-old pooled germinating seeds, were lysed with a buffer consisting of 4% sodium dodecyl sulfate, supplemented with 2× Thermo Halt protease inhibitor mixture in 100 mm HEPES, pH 7.5 for 10 min at 95 °C. Mechanical disruption was performed with single use pestles in protein Lobind tubes, followed by heating to 95 °C for 10 min and subsequent chilling on ice for 5 min. Proteomes were reduced with 5 mm DTT for 30 min at 56 °C, alkylated with 15 mm iodoacetamide (IAA) for 30 min in the dark at RT, and quenched by addition of additional 15 mm DTT and incubation for 15 min at RT.

##### SP3 Bead Binding and Proteome Clean Up

After reduction and alkylation, prepared SP3 beads were added to protein mixtures with a 1:10 ratio (w/w) protein/SP3 beads. Pure 100% ethanol was added to a final volume 80% v/v to initiate binding. After 18 min incubation at RT, supernatant was removed with assistance of a magnetic stand and the beads were rinsed two times with 400 μl 90% ethanol. Beads were resuspended by pipette mixing, with 30s break between each step to allow beads to settle on the magnetic stand. The remaining ethanol was spun down prior to the removal of supernatant and beads were resuspended in 30 μl 200 mm HEPES, pH 7.0.

##### Protein Dimethyl Labeling

2 m freshly prepared formaldehyde solution (Sigma-Aldrich, cat. no. 252549) and 1 m sodium cyanoborohydride (Sigma-Aldrich, cat. no. 296813) were added to 30 mm and 15 mm final concentration, respectively. In the *Arabidopsis* seedling experiment, ^12^CH_2_O formaldehyde was used for labeling of WT proteome and heavy ^13^CD_2_O formaldehyde (Sigma, cat. no. 596388) for the VPE0 quadruple mutant proteome. The lysate was incubated at 37 °C for 1 h in an oven, before repeated addition of fresh labeling reagents and incubation for another hour. To quench the reaction, 4 m Tris buffered to pH 6.8 (Fisher BioReagents, cat. no. BP153-1) was added to a final concentration of 600 mm (500 mm for *Arabidopsis* seedling proteome) and incubated at 37 °C for 3 h (30 min for *Arabidopsis* seedling proteome). For removal of excess reagents, new SP3 beads were added at a 1:5 ratio and protein bound by addition of 100% ethanol to a final concentration of 80% v/v ethanol. Beads were settled on a magnetic stand after 15 min incubation at RT, supernatant removed, and the beads washed twice with 400 μl of 90% ethanol. The tube was briefly centrifuged to collect and remove the remaining wash solution before resuspension of the beads in 30 μl trypsin in 200 mm HEPES buffer, pH 8.0. Beads were fully immersed in the solution and the trypsin to protein ratio was at least 1:100. After incubation at 37 °C in an oven for at least 13 h, 10% of the sample was removed to assess dimethyl labeling efficiency or to quantify protein abundance (pre-HUNTER sample). The reaction was mixed by tapping and 30s sonication after addition of each new reagent. Differentially labeled *Arabidopsis* seedling WT and VPE0 proteomes were pairwise combined after this step.

##### Enrichment of Protein N Termini by Undecanal-Assisted Negative Selection

100% ethanol was added to the proteome digest to 40% v/v before addition of undecanal (EMD Millipore, cat. no. 8410150025) at an undecanal/peptide ratio of 20:1 w/w (50:1 for *Arabidopsis* seedling samples) and addition of 1 m sodium cyanoborohydride to a final concentration of 30 mm. The pH was confirmed between pH 7–8 before incubation at 37 °C for 1 h. The reaction was sonicated in a water bath at 60kHz for 15 s and bound to magnetic rack for 1 min. The supernatant was transferred to a new Lobind tube and acidified with 0.5% trifluoroacetic acid (TFA) (Sigma-Aldrich, cat. no. T6508–100 ml) in 40% ethanol to pH 3–4 before loading onto a C18 column for removal of undecanal-tagged peptides. Different columns were chosen to provide enough binding capacity for excess undecanal reagent: Self-packed 4-layered C18 stage-tips were chosen for 1 to 5 μg protein; microspin column (Nest Group Inc, cat. no. S.E. SS18V) for 5 to 20 μg protein; macrospin column (Nest Group Inc, cat. no. SMM SS18V) for 20 to 100 μg protein; sep-pak columns (Waters, cat. no. WAT054960) for 100–1000 μg protein; HR-X (M) spin columns (Macherey-Nagel, cat. no. 730525) for experiments with *Arabidopsis* and rat brain proteome. The sample volumes were topped up with 0.1% TFA in 40% ethanol to a loading volume was 80 μl, 200 μl, 400 μl and 500 μl for stage-tips, microspin, HR-X (M) spin, macrospin column, and sep-pak respectively. Before loading the samples, the stage-tips were conditioned with 100 μl methanol and followed by 100 μl 0.1% TFA in 40% ethanol whereas microspin column, macrospin column, HR-X (M) spin columns and sep-pak were conditioned with a volume of 200 μl, 200 μl, 400 μl and 700 μl respectively. After the conditioning of C18 columns, the samples were then loaded and the flow-through was collected in 1.5 ml protein Lobind tubes. The ethanol in the collected flow-through was removed by vacuum supported evaporation, peptides were resuspended in. 0.1% TFA in HPLC water and desalted using home-made C18 stage-tips or commercial reverse-phase C18 spin columns.

##### Automated HUNTER

Human peripheral blood plasma (STEMCELL Technologies, cat. no. 70039) and plasma and bone marrow interstitial fluid samples from three pediatric B-ALL patients (B-ALL-1, -2, -3) were processed on an epMotion M5073 automated liquid handling system (Eppendorf) controlled by an EasyCon tablet (Eppendorf). The HUNTER protocol was programed with epBlue Studio (ver. 40.4.0.38). The M5073 was configured with: dispensing tool TS50 (1.0–50 μl) and TS1000(40–1000 μl), epT.I.P.S. Motion racks (1.0–50 μl and 40–1000 μl), epMotion gripper, Thermoadapter for 96-PCR plate (skirted), Alpaqua Magnum FLX 96 magnet plate, Eppendorf rack for 24× safe lock, Twin.tec PCR plate 96 (semi-skirted; max. well volume is 250 μl).

The following adaptations to the HUNTER protocol were made to achieve optimal automation: 250–300 μg protein (maximum 5 μl plasma) was processed. Dimethylation was performed at room temperature, the final concentration of formaldehyde was 35 mm, and the final concentration of sodium cyanoborohydride was 15 mm. 2 units of benzonase were added to 5 μl of plasma. Wash steps were programmed to aspirate 10 μl more than the dispense volume to ensure full removal of all wash buffers. During the digestion and undecanal labeling steps, the plate was covered with thermal adhesive sealing film (Diamed Lab Supplies Inc., cat. no. DLAU658–1) and incubated at 37 °C. Samples and/or beads were mixed on the heater/shaker at 1500rpm for 2 min. To prevent bubbles forming in tips and ensure uniform dispensing, the aspiration speed was set to 10 mm/s. All pipetting steps were programmed to aspirate from bottom and dispense from top. Undecanal and ethanol were combined first before dispensing into each well.

##### Preparation of Single-pot Solid-phase-enhanced Sample Preparation (SP3) Beads

1:1 v/v ratio of hydrophilic (conc. 10 μg/μl, GE Life Sciences, cat. no. 4515–2105-050250) and hydrophobic Sera-Mag SpeedBeads carboxylate-modified magnetic beads (conc. 10 μg/μl, GE Life Sciences, cat. no. 6515-2105-050250) were combined in a 1.5 ml flex tube (Eppendorf, cat. no. 022364111), then place them on a magnetic stand (Life Technologies, cat. no. 12321D) for removal of supernatant. The beads were washed twice and reconstituted in HPLC water (Fisher Scientific, cat. no. W6–4) and stored at 4 °C.

##### Fluorometric and Colorimetric Protein and Peptide Measurements

To evaluate labeling, binding and elution efficiencies during protocol optimization, peptide concentration and primary amine reactivity were quantified using the Pierce quantitative fluorometric peptide assay (Thermo Fisher Scientific, cat. no. 23290) and Pierce quantitative colorimetric peptide assay (Thermo Fisher Scientific, cat. no. 23275) following the assay protocols.

##### Optimizing Dimethyl Labeling of Proteins

Ten micrograms of reduced and alkylated HeLa protein were used as starting material. 2 m fresh formaldehyde and 1 m sodium cyanoborohydride were added to 30 mm and 15 mm final concentration in 200 mm HEPES, pH 7.0. The reaction was incubated at 37 °C as indicated. After the first incubation, fresh labeling reagents were added and incubated at 37 °C for 1 h. Both LC-MS/MS analysis and the amine-reactive quantitative fluorometric peptide assay were performed to evaluate the dimethyl labeling efficiency.

##### Optimizing Undecanal Modification of Peptide α-Amines

Frozen plant leaves or rat brains were homogenized in 6 m GuHCl, 0.1 m HEPES pH 7.4, 1 mm DTT, 5 mm EDTA and 1× Thermo Halt Protease inhibitor mix with a Kinematica Polytron PT-2500 for 2 × 30 s at 18,000 rpm (Kinematica, Luzern, Switzerland). Homogenate was filtrated through Miracloth (Merck, Darmstadt, Germany) and cell debris pelleted at 500 g for 5 min, 4 °C. Supernatants were chloroform/methanol precipitated, resuspended in 1:2 diluted homogenization buffer, reduced by incubation with 5 mm DTT for 30 min at 56 °C and alkylated by addition of 15 mm IAA for 30 min in the dark at RT. The reaction was quenched by addition of additional 15 mm DTT and incubation for 15 min at RT.

Dimethylation was performed at protein level with 20 mm heavy formaldehyde (^13^CD_2_O) and 20 mm sodium cyanoborohydride at 37 °C O/N. The next day, fresh 20 mm formaldehyde and sodium cyanoborohydride were both added for a further dimethylation of 2 h at 37 °C. Labeled proteins were purified by chloroform-methanol precipitation and concentration determined using the BCA assay (BioRad). Samples were digested over night at 37 °C with MS-grade trypsin (Serva) at a 1:100 protease/protein ratio in digestion buffer (0.1 m HEPES pH 7.5, 5% ACN, 5 mm CaCl_2_). Digestion was prolonged by addition of fresh MS-grade trypsin at a 1:200 ratio for 2 h at 37 °C.

Trypsin-generated peptide α-amines were hydrophobically modified by adding undecanal in a 50:1 (w/w) ratio undecanal/proteome and 20 mm sodium cyanoborohydride in 40% ethanol (final concentration). The reaction was incubated at 50 °C for 45 min, followed by addition of 20 mm sodium cyanoborohydride and further incubation for 45 min. The reaction was quenched by acidification with 1% TFA. Supernatants were depleted of undecanal and undecanal-modified peptides using HR-X (M) cartridges (Macherey-Nagel, Dueren, Germany). Briefly, cartridges were activated by 2 ml 100% ACN, washed with 2 ml 2% ACN + 0.1% TFA and samples were loaded once on the cartridge and the flow-through containing dimethyl-blocked terminal peptides was collected. To elute remaining dimethyl-blocked terminal peptides from the cartridge, a second elution with 1 ml 40% ACN + 0.1% TFA was performed and the combined flow-through was evaporated in a SpeedVac to a small sample volume which was desalted and purified by C18 stage-tips.

##### Optimizing Undecanal Removal

In this experiment, the removal of undecanal with 40%/50% ethanol and acetonitrile using three different C18 columns was tested. 412 μg, 1650 μg, and 8250 μg undecanal in 0.1% TFA in 40%/50% ethanol and acetonitrile were spun through fully conditioned stage-tip, microspin column, or sep-pak C18 columns respectively. The flow-through was collected in 1.5 ml Eppendorf tubes and the volume reduced in a SpeedVac. 10 μg HeLa peptides and 1 m sodium cyanoborohydride were added to a final concentration of 30 mm. The volume was adjusted with 40% ethanol to a final volume of 20 μl. The samples were incubated at 37 °C for 1h and then measured using the quantitative fluorescent peptide assay. The undecanal calibration curve was constructed from 0 μg/μl to 41.3 μg/μl.

##### Evaluation of Peptide Recovery Dependence on Solvent Concentrations

Stage-tips with 4 C18 disks were prepared and conditioned with methanol and 0.1% TFA in water. 10 μg HeLa peptides were loaded on stage-tips and centrifuged at 1200g. The peptides were sequentially eluted with 40% ethanol, 50% acetonitrile, and 80% acetonitrile and collected in 1.5 ml Eppendorf tubes. Then, the samples were dried with speed vac and topped up with water to 10 μl. The samples were sonicated before performing colorimetric peptide quantification. Elution with 80 and 100% acetonitrile respectively and initial HeLa peptides were used as controls.

### Mass Spectrometry

#### 

##### Data-dependent Acquisition (DDA)

Pre-HUNTER and post-HUNTER HeLa and clinical samples were analyzed on a Q Exactive HF plus Orbitrap mass spectrometer coupled to an Easy-nLC 1200 liquid chromatography (Thermo Scientific) with a 3 cm-long homemade precolumn (Polymicro Technologies capillary tubings, 360OD, 100ID), a 35 cm-long homemade analytical column (Self-pack PicoFrit columns, 360OD, 75ID, 15 μm tip ID) and packed with Dr. Maisch beads (ReproSil-Pur 120 C18-AQ, 3 um) with a flow rate at 300 nL/min and constant temperature at 50 °C. Mobile phase A (0.1% formic acid in water) and mobile phase B (0.1% formic acid in 95% acetonitrile) were used for a 65 min gradient (3–8%B in 3 min, 8–27%B in 37 min, 27–42%B in 12 min; 42–100%B in 13 min). DDA: A full-scan MS spectrum (350–1600 *m*/*z*) was collected with resolution of 120,000 at *m*/*z* 200 and the maximum acquisition time of 246 ms and an AGC target value of 1e6. MS/MS scan was acquired at a resolution of 60,000 with maximum acquisition time of 118 ms and an AGC target value of 2e5 with an isolation window of 1.4 *m*/*z* at Orbitrap cell. The top 12 precursors were selected. Normalized collision energy (NCE) was set to 28. Dynamic exclusion duration was set to 15 s. Charge state exclusion was set to ignore unassigned, 1, and 5 and greater charges. The heated capillary temperature was set to 275 °C. It should be noted that 0.8 μg peptides in plasma samples, 1 μg peptides in 500 K and 1 m HeLa post-HUNTER samples and all peptides in 10 K, 20 K, and 100 K HeLa post-HUNTER samples were injected for LC-MS/MS analysis.

*Arabidopsis* leaf and rat brain samples were analyzed on a two-column nano-HPLC setup (Ultimate 3000 nano-RSLC system with Acclaim PepMap 100 C18, ID 75 μm, particle size 3 μm columns: a trap column of 2 cm length and the analytical column of 50 cm length, ThermoFisher) with a binary gradient from 5–32.5% B for 80 min (A: H_2_O + 0.1% FA, B: ACN + 0.1% FA) and a total runtime of 2 h per sample coupled to a high resolution Q-TOF mass spectrometer (Impact II, Bruker, Bremen, Germany) as described (16). Data was acquired with the Bruker HyStar Software (v3.2, Bruker) in line-mode in a mass range from 200–1500 m/z at an acquisition rate of 4 Hz. The Top17 most intense ions were selected for fragmentation with dynamic exclusion of previously selected precursors for the next 30 s unless intensity increased 3-fold compared with the previous precursor spectrum. Intensity-dependent fragmentation spectra were acquired between 5 Hz for low intensity precursor ions (> 500 cts) and 20 Hz for high intensity (> 25k cts) spectra. Fragment spectra were collected using two parameter sets, each, with 50% of the acquisition time: 61 μs transfer time, 7 eV collision energy and a collision RF of 1500 Vpp followed by 100 μs transfer time, 9 eV collision energy and a collision RF of 1800 Vpp.

##### Data-independent Acquisition (DIA)

The samples were resolubilized in 0.1% formic acid and spiked with iRT peptides before analysis on the Q-Exactive HF system (Thermo) described above. For 1 million *HeLa* samples (1 μg of protein was injected), a full-scan MS spectrum (350–1650 *m*/*z*) was collected with resolution of 120,000 at *m*/*z* 200 and the maximum acquisition time of 60 ms and an AGC target value of 3e6. DIA segment spectra were acquired with a twenty-four-variable window format with a resolution of 30,000 with an AGC target value of 3e6, and using 25% normalized collision energy (NCE) with 10% stepped NCE. The stepped collision energy was 10% at 25% (NCE = 25.5 - 27.0 − 30.0). The maximum acquisition time was set to “auto.” DIA method for 20,000 *HeLa* samples was slightly adjusted to accommodate low complexity samples. A 10-variable window format was applied with a resolution of 60,000 and an AGC target of 3e6. The stepped collision energy (NCE) was 28. A default charge state of 3 was applied for MS2 acquisition scans.

##### Data Processing

Raw MS DDA data acquired on the Q Exactive HF were processed and searched with MaxQuant (28) version 1.6.2.10 using the built-in Andromeda search engine. The first search peptide tolerance of 20 ppm and main search peptide tolerance of 4.5 ppm were used. The human protein database was downloaded from UniProt (release 2018_09; 20,410 sequences) and common contaminants were embedded from MaxQuant. The “revert” option was enabled for decoy database generation. For analysis of enriched N termini (post-HUNTER) samples, dimethyl (peptide N-term and K) were selected as fixed modifications whereas oxidation (M), acetyl (N-term), Gln→pyro-Glu, and Glu→pyro-Glu were dynamic modifications. ArgC semispecific free N terminus digestion with maximum two missed cleavage sites. The label free quantification minimum ratio count was 1. “Match between runs” was only enabled for clinical samples. The false discovery rate for PSM, peptide and protein were set as 1%. Label-free quantification was used to quantify the difference in abundance of N termini between samples. To determine dimethyl labeling efficiency and pullout efficiency from pre- and post-HUNTER samples respectively, oxidation (M), acetyl (N-term), dimethyl (K), dimethyl (N-term), Gln→pyro-Glu, and Glu→pyro-Glu were selected as dynamic modifications. ArgC specific digestion mode was used in the first search and Trypsin/P semi-specific digestion mode was selected in the main search. To calculate pullout efficiencies dimethyl (peptide N-term) was defined as variable modification and to calculate labeling efficiencies both dimethyl (peptide N-term and K) were set as variable modifications.

*Arabidopsis* and rat brain DDA data acquired with Impact II Q-TOF instruments were processed and searched with MaxQuant (28) v.1.6.3.3 using embedded standard Bruker Q-TOF settings that included peptide mass tolerances of 0.07 Da in first search and 0.006 Da in the main search. The *Arabidopsis* and rat protein databases were downloaded from UniProt (*Arabidopsis*: release 2018_01, 41350 sequences; rat: release 2017_12, 31571 sequences) with appended common contaminants as embedded in MaxQuant. The “revert” option was enabled for decoy database generation. Database searches were performed as described above, except that enzyme specificity was set as Arg-C semi specific with free N terminus also in the first search, heavy dimethylation with ^13^CD_2_O formaldehyde was set as label (K) whereas oxidation (M), acetyl (N-term), heavy dimethyl (N-term), Gln→pyro-Glu, and Glu→pyro-Glu were set as dynamic modifications. Data analysis of the *Arabidopsis* seedling experiment considered duplex dimethyl labeling with light ^12^CH_2_O formaldehyde or heavy ^13^CD_2_O formaldehyde (peptide N-term and K).

DIA was analyzed with Spectronaut Pulsar X (version 12.0.20491.0.21112, Biognosys, Schlieren, Switzerland). First, a spectral library was generated by searching the DIA raw files for samples together with 36 DDA files acquired on 12 high-pH fractions for triplicate HeLa samples in Spectronaut Pulsar. The default settings were applied with the following changes: Digest type was semi-specific (free N terminus) for Arg C, minimum peptide length = 6. Carbamidomethyl (C) and dimethyl (K) were fixed modifications, whereas variable modifications consisted of oxidation (M), acetyl (N-term), dimethyl (N-term), Gln→pyro-Glu, and Glu→pyro-Glu. The resulting spectral library contained precursor and fragment annotation and normalized retention times. This was used for targeted analysis of DIA data using the default Spectronaut settings. In brief, MS1 and MS2 tolerance strategy were “dynamic” with a correction factor of 1. Similar setting was maintained for the retention time window for the extracted ion chromatogram. For calibration of MS run precision iRT was activated, with local (non-linear) regression. Feature identification was based on the 'mutated' decoy method, with dynamic strategy and library size fraction of 0.1. Precursor and protein false discovery rate were 1% respectively. The report generated from Spectronaut was filtered for N-terminal peptides with dimethyl and acetyl modifications.

##### Data and Statistical Analysis

Data evaluation and positional annotation was performed using an in-house Perl script that combines information provided by MaxQuant, UniProt and TopFINDer (29) to annotate and classify identified N-terminal peptides. The script (manti.pl) is publicly available (http://manti.sourceforge.io) and will be presented in detail elsewhere. In short, MaxQuant peptide identifications are consolidated by removing non-valid identifications (peptides identified with N-terminal pyro-Glu peptides that do not contain Glu or Gln as N-terminal residue, peptides with dimethylation at N-terminal Pro), contaminant, reverse database peptides, and non-quantifiable acetylated peptides in multi-channel experiments (no K in peptide sequence to determine labeled channel). For peptides mapping to multiple entries in the UniProt protein database, a “preferred” entry was determined by selecting protein entries where the identified peptide matches position 1 or 2, then manually reviewed UniProt protein entries are favored. If multiple entries persisted, the alphabetically first was chosen by default. For *Arabidopsis* seedling experiments, changes in peptide abundance were tested for significance as previously published (30) using the LIMMA-moderated *t* test as implemented in the R limma package. Abundance changes greater than 2-fold (log2 <−1 or >1) associated with a *p* value < 0.05 were considered significant.

Proteins identified in human plasma before and after N termini enrichment were annotated with their previously reported plasma protein concentration (31). N-terminal peptides identified from mitochondria were compared with recently reported N termini identified in HeLa cells (32) and listed in the MitoCarta2.0 database (33). Cleavage site patterns surrounding identified mitochondrial N termini or altered protease-generated N termini in the *Arabidopsis* seedlings were visualized as iceLogo (34) (https://iomics.ugent.be/icelogoserver/) and WebLogo (https://weblogo.berkeley.edu/logo.cgi). Supplemental Table S1 links analyses and figures with the underlying data found in supplemental Tables S2–12 and Data sets 1–11. Raw data has been deposited as stated.

##### Label Free Quantification

For label free quantification muda.pl pre-processed data with peptide intensities determined by MaxQuant, is processed further by eliminating termini with intensity values for <20% of the analyzed samples. Data is median normalized, followed by multiplication by the overall data median and log(10) transformation. Pearson correlations, Coefficients of Variation and LIMMA-moderated *t* test *p* values are calculated using standard implementations in R or python. To retain sample specific termini, missing values were imputed with values randomly selected from a distribution modeled after the tenth to twentieth percentile of the whole data and down-shifted by a random factor of 50–100 placing imputed values into the very low intensity area of the data. Radar plots display the z-score standardized intensity on the *y* axis, and fuzzy c-means cluster membership encoded as the line color. Radar plots and t-distributed stochastic neighbor embedding (t-SNE) followed by fuzzy clustering based on imputed data are used for unsupervised characterization of relationships.

## RESULTS AND DISCUSSION

### 

#### 

##### Workflow Optimization

**Fig. 1.**
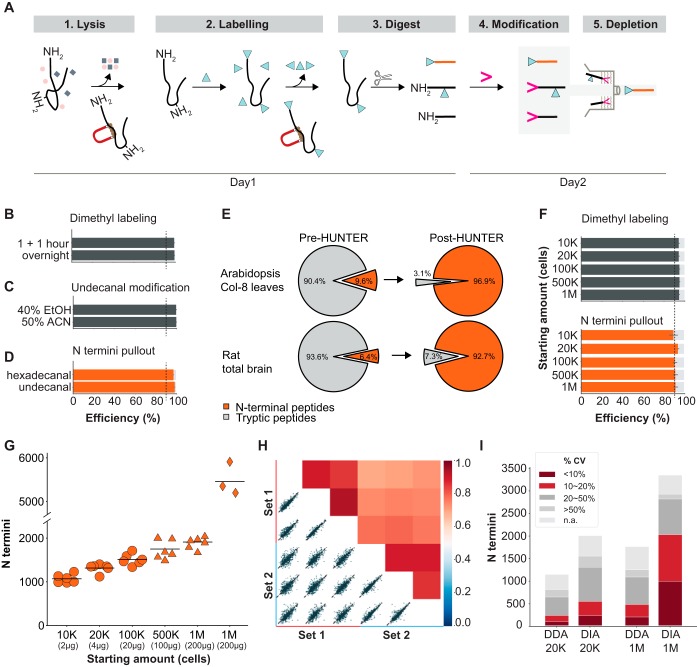
**A protocol for sensitive enrichment and identification of N-terminal peptides.** *A*, Workflow: (1) Lysis and protein purification with SP3 magnetic beads; (2) modification of N-terminal α- and lysine ε-amines; (3) proteome digestion; (4) modification of digestion-generated peptide α-amines with undecanal in 40% ethanol (gray background); (5) retention of undecanal-modified peptides on reverse-phase columns, whereas N-terminal peptides pass through. *B–D*, Optimization of individual steps. *B*, Completeness of on-bead dimethyl labeling of HeLa cell lysates (*n* = 3 technical replicates). *C*, Degree of HeLa-derived peptide undecanal modification in organic solvents, assessed by reactivity of unlabeled amines (*n* = 3 technical replicates). *D*, Comparison of modification reagents -hexadecanal and undecanal- assessed by N termini enrichment from *Arabidopsis thaliana* leaf proteome (*n* = 3 biological replicates). *E*, Fraction of N-terminal and digest-generated peptides in *Arabidopsis* whole leaf and rat total brain proteome digests before (pre-HUNTER) and after (post-HUNTER) enrichment. Shown are average values from three independent biological replicates. *F–H*, Evaluation of HUNTER workflow using varying starting amounts of HeLa cells. *F*, Dimethyl labeling efficiency assessed by lysine side chain modification before depletion (top) and N termini enrichment efficiency after enrichment (bottom). *G*, Number of N termini identified from 10,000 to 1,000,000 HeLa cells. Circles, samples with <1 μg peptide analyzed in a single injection; triangles, larger samples with 1 μg enriched peptides analyzed per replica; diamonds, with offline high-pH pre-fractionation. Datapoints represent technical replicates, lines indicate the mean. *H*, Intensity-based Pearson-correlation between two manually processed N termini enrichment datasets from 20,000 HeLa cells, identified by data-dependent acquisition (DDA, each set *n* = 3 technical replicates). *I*, Comparison of coefficients of variation (CV) for N termini from 20K and 1 m HeLa cell starting material quantified by label free DDA or DIA. Average CV of pairwise comparisons of quantified terminal peptides in triplicate analysis is displayed. NAN denotes termini quantified in only one replica. All error bars indicate S.D.

The most sensitive protocols established to date enrich protein N termini by negative selection, where protein amines are blocked with amine-reactive reagents before proteome digestion (24). This in turn generates new peptide-N-terminal α-amines that are then exploited for depletion. Unspecific losses leading to low reproducibility mainly occur in three critical steps (Fig. 1*A*, supplemental Fig. S1*A*), removal of free amino acids and other interfering compounds from the protein lysate, removal of amine-reactive labeling reagents prior to digestion and selective depletion of proteome digestion-generated non-N-terminal peptides. To overcome this, we replaced protein precipitation as the common proteome purification procedure by reversible high-efficiency binding to hydroxylated magnetic beads as used in the Single-Pot Solid-Phase-enhanced Sample Preparation (SP3) method (35, 36). We first established compatibility of SP3 with protein-level dimethyl labeling and found that within 2 h >99% of primary amines on proteins were successfully blocked from subsequent reaction (Fig. 1*B*). The third loss-intensive step is depletion, where unspecific binding of dilute N-terminal peptides to surfaces of filters, beads and other consumables are particularly problematic for microscale samples. Here we adapted a strategy based on attaching hydrophobic hexadecanal to the free peptide α-amines generated by the proteome digest (12). This increased the hydrophobicity of tryptic non-N-terminal peptides, allowing their retention on a reverse phase liquid chromatography column, whereas N-terminal peptides were eluted and directly analyzed by MS/MS. However, hexadecanal-containing reactions solidified at room temperature and underwent phase separation resulting in losses and lowered reproducibility. We tested the shorter-chain undecanal, which is liquid at room temperature. After optimizing reaction time (supplemental Fig. S1*B*, S1*C*) and solvent conditions (Fig. 1*C*), we found that reaction in 40% ethanol for 60 min at 37 °C, followed by passing the reaction mixture through commercial C18 reverse phase resins (supplemental Fig. S1*D*) allowed direct enrichment with minimal loss of N-terminal peptides (supplemental Fig. S1*E*). This depletion of undecanal-tagged peptides was equally or more efficient compared with hexadecanal (Fig. 1*D*, supplemental Fig. S2*A*, S2*B*), resulting in enrichment of N-terminally modified peptides from baseline levels of <10% to >92% after enrichment. The enrichment efficiency was independent from the amount and source of digested proteome used for pullout, including *Arabidopsis thaliana* leaf and rat brain proteomes (Fig. 1*E*, supplemental Fig. S2*C*, S2*D*).

##### Performance Assessment

After optimizing SP3 based labeling and undecanal-mediated depletion individually, we evaluated the performance of the combined workflow in a one-pot reaction from lysis to cleanup. Across a wide range of starting material from 1 million HeLa cells, equivalent to 200 μg protein lysate, down to as few as 10,000 HeLa cells, or 2 μg crude protein lysate, we observed >94% dimethyl-modified lysine residues and enrichment efficiencies >90% (Fig. 1*F*). Analysis with an Orbitrap Q-Exactive HF mass spectrometer identified an average of 1057, 1230 and 1454 N-terminal peptides from 2 μg, 4 μg and 20 μg crude protein lysate, respectively, within one hour. For larger samples, only 1 μg of the recovered N-terminal peptides were injected, resulting in the identification of 1810 N-terminal peptides on average. High-pH fractionation of N-terminal peptides enriched from 200 μg HeLa proteome readily increased this to identification of >5000 N-terminal peptides (Fig. 1*G*).

We next evaluated if the gain in sensitivity compared with previous studies is largely because of improved instrumentation or can be attributed to improvements in the sample preparation. To this end we performed two independent experiments for which we processed duplicates of 10,000, 100,000, 500,000, and 1 million HELA cell pellets each by HUNTER and Terminal Amine Labeling of Substrates (TAILS) (9) in parallel. Among several established methods for N termini enrichment, (24) we chose TAILS for this comparison because we have long-standing experience in this extraordinarily successful and proven approach (14, 16, 37, 38). Our comparison showed that HUNTER enabled identification of hundreds of N termini in small amounts of starting material (2 and 20 μg respectively) that were not amenable to TAILS analysis (<100 termini identified on average). In contrast, for starting amounts larger than 500,000 HELA cells (100 μg crude lysate) the identification rates were comparable between both methods (supplemental Fig. S3*A*). Individual HUNTER and TAILS replica show slightly lower (25%) overlap in identical N termini identification compared with those shared between two HUNTER (32%) or two TAILS (35%) replica (supplemental Fig. S3*B*). The observed overlap between two HUNTER or TAILS replica is in line with other reports (25) and indicated strong under-sampling in our short DDA analyses. Remarkably, the overlap between HUNTER and TAILS replica was very similar and N termini identified by TAILS and HUNTER were very similar in length (supplemental Fig. S3*C*), molecular mass (supplemental Fig. S3*D*), peptide and associated protein hydrophobicity (supplemental Fig. S3*E*, S3*F*). This suggested that there is no strong bias in the N termini identified by HUNTER as compared with TAILS.

To assess whether the N termini identified at low starting amounts are indeed true identifications, we evaluated the percentage of N termini observed uniquely in these datasets. We hypothesized that most genuine N termini identified from small starting amounts should also be identified in the more comprehensive analyses from larger starting amounts. Indeed, only 2% (35) or 8% (177) of N termini were uniquely identified from 2 or 4 μg of crude lysate but not in larger scale HUNTER experiments (supplemental Fig. S4*A*), underscoring that also low starting amounts yield predominantly high-confidence N termini identifications.

After confirming that the new experimental workflow enabled reduction of the input material by up to fifty-fold we explored if further improvements could be gained by employing modern MS/MS acquisition approaches. Data independent acquisition (DIA) using a spectral library extracted from these fractions further boosted the number of N termini identified from 200 μg starting material in a single 1-hour analysis by 50% to 2877 (supplemental Fig. S4*B*). The reproducibility was similar across the range of starting material, with Pearson correlation factors of 0.89 between manually pipetted replica of 4 μg HeLa material and 0.74 between days (Fig. 1*H*, supplemental Fig. S4*C*–S4*F*), as reported for label-free single-peptide quantification with minimal starting amounts (27). DIA analysis of 4 μg and 200 μg HeLa lysate showed similar correlation coefficients of 0.91 between manually pipetted replica (Supplemental Fig. S4*G*, S4*H*), but markedly improved quantitative precision. DIA analysis resulted in a 5-fold increase of N termini quantified at CVs <10%. By DIA 988 N termini were quantified in 200 μg with single peptide CVs <10%, whereas DDA analysis only quantified 192 N termini with CVs <10%. DIA further allowed quantification of more N termini at CVs <20% than the total number of termini identified by DDA (Fig. 1*I*). With the new protocol in place, we set out to explore the performance and utility of the HUNTER protocol with samples that had so far not been amenable to N terminome characterization.

##### Application I - N terminome Profiling in Sorted Cells

**Fig. 2.**
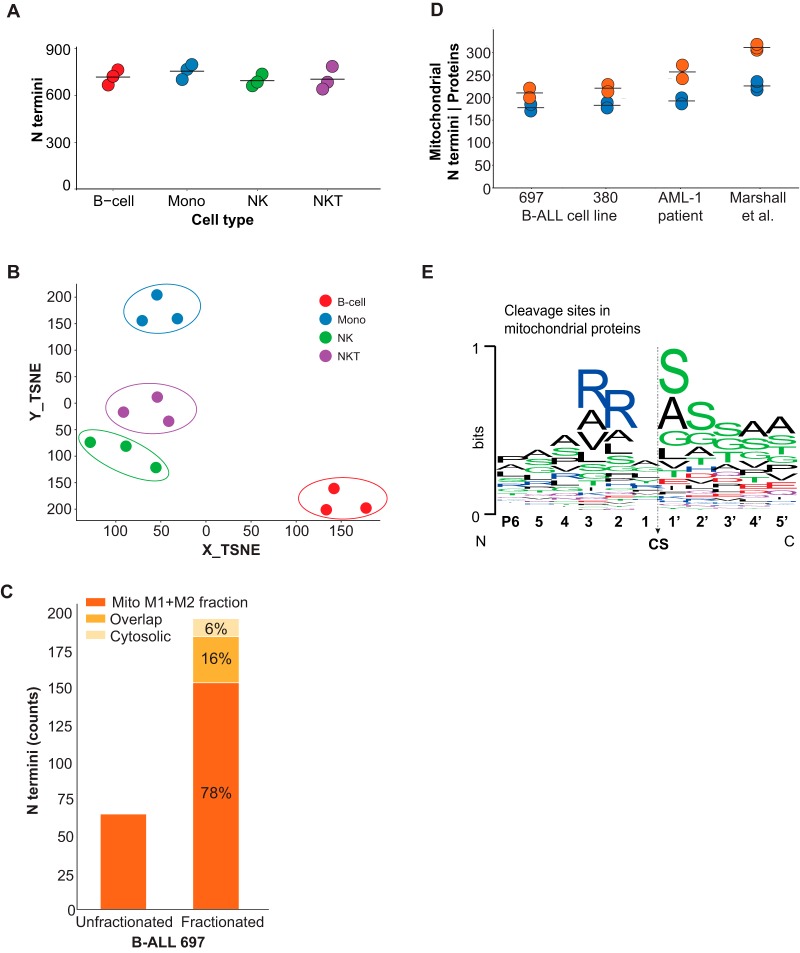
**N terminome analysis in sorted cells and subcellular compartments.** *A–B*, N terminome analysis of sorted human peripheral blood mononuclear cells (PBMC). A, N termini identified from 30,000 B-cells, monocytes, natural killer (NK) cells and NKT-cells, mean indicated by line. (DDA, *n* = 3 technical replica) *B*, Unsupervised t-distributed stochastic neighbor embedding (t-SNE) based on raw log_10_ transformed N-terminal peptide intensities. *C–E*, Subcellular N terminome. C, Evaluation of subcellular enrichment efficiency for B-ALL cell line 697. Total number of identified mitochondrial termini before and after crude subcellular fractionation of 2.5 million cells in 30 μl buffer using pressure cycling technology (PCT) assisted lysis. *D*, Mitochondrial protein N termini enriched from 2.5 million B-ALL 697, B-ALL 380 and AML patient cells compared with N termini identifications from 12–15 million HeLa cells as reported by Marshall *et al.* Orange, N termini; Blue, proteins. *E*, Consensus sequence logo of 233 mitochondrial N termini identified from AML-1 patient cells. The logo is dominated by sequences matching the sequence specificity of the classical mitochondrial transit peptide cleavage site.

The ability to obtain deep N terminome data from less than 1 million cells facilitates the investigation of specific cell populations rather than bulk mixtures. One classic example of a complex mixture of highly specialized cell types are blood monocytes. Many cell types have far lower protein content compared with HELA cells we used to establish the HUNTER protocol and often occur at frequencies below 10% or even below 1% in human blood. To enable replica analyses and avoid collection of excessive volumes of blood (1 ml blood contains about 1–2 million monocytes), methods that require fewer than 100,000 cells per analysis are required. To demonstrate the ability of HUNTER to study genome encoded and proteolysis generated termini and their differences between human peripheral blood monocyte types, we isolated B-cells, monocytes, NK-cells and NKT-cells by fluorescence-activated cell sorting. For each population we analyzed triplicates of only 30,000 sorted cells and identified between 646 and 803 N termini (Fig. 2*A*). Unsupervised dimensionality reduction based on N termini abundance clearly separated the different cell types, with replica of each cell type grouped in close proximity (Fig. 2*B*), demonstrating specificity of N termini profiles. Interestingly we could not observe any significant cell-type specific differences in N-terminal acetylation (supplemental Fig. S5*A*), degree of internal protein processing or protease activities.

##### Application II - N Termini Identification in Subcellular Compartments

Next, we asked if HUNTER could support investigation of proteolytic processes in subcellular compartments of limited samples, such as pediatric patient biopsies. Many biological processes are strictly confined to a specific subcellular compartment and analysis of whole cell homogenates may mask the effect. This type of study was previously restricted to cultured cells of which large quantities of source material could be obtained. We first optimized a crude subcellular fractionation of mitochondria using mild pressure cycling assisted cell lysis of 2.5 million cells in 30 μl buffer, resulting in a 3-fold increase of N termini originating from known mitochondrial proteins (Fig. 2*C*). HUNTER applied to mitochondrial fractions from less than 2.5 million Acute Myeloid Leukemia (AML) blasts obtained by bone marrow aspiration from a pediatric patient enabled detection of 257 N termini for 193 mitochondrial proteins, with similar numbers identified in two B-ALL cell lines (supplemental Fig. S5*B*, S5*C*). Compared with a recent study of mitochondrial protein processing in human cells (32), HUNTER identified on average 73% of the mitochondrial protein termini and 81% of mitochondrial proteins from only about 1/10th of the starting material (Fig. 2*D*). An alignment of the mitochondrial N termini matched the previously described pattern resulting from transit peptide cleavage and subsequent aminopeptidase processing of nuclear-encoded proteins imported into mitochondria (Fig. 2*E*) (30).

##### Application III - Protease Substrate Identification

**Fig. 3.**
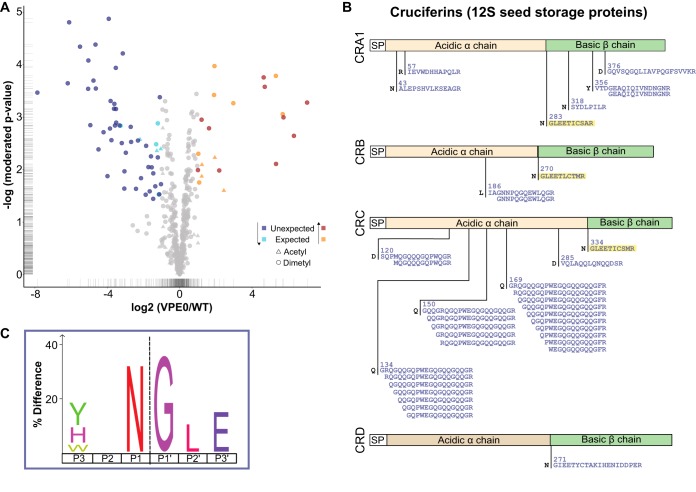
**Seed storage protein processing in *Arabidopsis* seedlings.** Comparative N terminome analysis of 2.5-day old *Arabidopsis* VPE0 quadruple mutants and WT seedlings. *A*, Volcano plot of four biological replicate experiments with three pooled seedlings per genotype. Circle, dimethylated N termini; triangle, acetylated N termini; light blue/orange, significantly less/more abundant (*p* value <0.05 with log_2_(VPE0/WT)<−1 or >1) N-terminal peptides matching known or predicted protein N termini; dark blue/dark red, significantly less/more abundant (*p* value <0.05, log_2_<−1 or >1) neo-N termini matching “unexpected” positions within the protein models. *B*, Protein N termini derived from 12S seed storage proteins with reduced abundance in the VPE deficient mutant. Cleavage sites previously identified by Edmann sequencing are highlighted in yellow. *C*, iceLogo representing 24 cleavage sites deduced from 47 unexpected neo-N-terminal peptides that are significantly more abundant in wt, filtered for redundancy created by exopeptidase ragging. Dashed line indicates cleavage site.

Protease substrate identification is one of the key applications of N terminome analyses providing unbiased mechanistic insights into protease function that cannot be obtained otherwise. To test the utility of HUNTER for substrate identification in small specimens, we chose to compare *A. thaliana* wild type seedlings with a quadruple mutant lacking all four genes coding for vacuolar processing enzymes (VPEs). It is well known that 12S globulin and 2S albumin seed storage proteins accumulate with different processing/maturation patterns in seeds of VPE-deficient plants (39, 40). We therefore reasoned that these mutants would be an ideal model to test substrate identification with HUNTER. We first analyzed differentially stable isotope labeled protein extracts from single wild-type and VPE-deficient seedlings, but challenging proteome extraction and the high biological variation in early development resulted in the identification of only few consistent changes that notably included N termini of 12S seed storage proteins that only accumulated in wild type but not in the mutant (supplemental Fig. S6). To account for biological variability, facilitate proteome extraction and increase coverage, we then pooled three 2.5-day-old seedlings per condition (supplemental Fig. S7). HUNTER analysis with stable isotope labeled samples enabled identification of 900 N-terminal peptides. Of the 500 N-terminal peptides quantified in two or more of the four replicate experiments, 75 N termini showed significant differences (*t* test *p* value <0.05, at least 2-fold change in abundance) between both lines (Fig. 3*A*). 54 N termini were more abundant in wild-type than in the mutant (Fig. 3*A*), most of which reflected altered processing of 12S seed storage proteins (Fig. 3*A*) including several known VPE-dependent 12S seed storage protein maturation sites (39, 40). Many of the observed VPE-dependent cleavages resulted in ragged termini, *i.e.* a series of sequentially truncated N termini, particularly in Gln-rich stretches (Fig. 3*B*), which may indicate a mechanism for controlled mobilization of nitrogen from seed storage proteins. To account for such a potential Gln-specific aminopeptidase activity, we further filtered for the longest representative peptide at each cleavage site resulted in 24 N termini that predominantly matched the known VPE sequence specificity for cleavage after Asn (Fig. 3*C*) (41, 42). The 21 N-terminal peptides with increased abundance in the VPE null mutant indicated alternative processing of 2S seed storage proteins, preferentially between Glu or Trp and Phe (supplemental Fig. S7*D*, S7*E*). Six of these N-terminal peptides have been previously identified by Edman sequencing of selected protein bands after SDS-PAGE separation of proteome extracts from hundreds of pooled seeds (Fig. 3*B*) (39, 40). The alternative processing of seed storage proteins in VPE-deficient seeds has been linked to aspartyl protease activity (40), which is also supported by the overrepresentation of large hydrophobic residues at the cleavage site (supplemental Fig. S7*D*). In addition, we observed an increased abundance of N-terminal peptides mapping to the activation sites of cathepsin B3 and the germination-specific cysteine proteases CP1 in the mutant, which might contribute to the altered seed storage protein processing pattern and help to compensate for the lack of VPE activity (supplemental Fig. S7*F*).

##### Application IV - Automated Liquid Biopsy Analysis

**Fig. 4.**
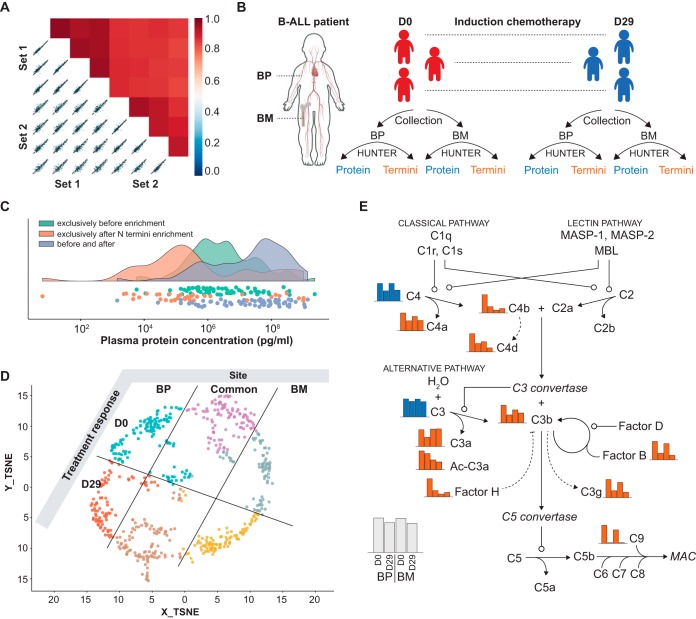
**Automated HUNTER analysis liquid biopsies.** *A*, Evaluation of technical robustness and reproducibility of the implemented automation. Intensity-based Pearson-correlation between two automatically processed sets (each *n* = 4 technical replicates) from human plasma samples, acquired by DDA. *B*, Study design. Blood plasma (BP) and bone marrow interstitial fluid (BM) was collected from three pediatric B-ALL patients at diagnosis (D0) and after induction chemotherapy (D29). All samples were processed by automated HUNTER and pre-enrichement (total protein) and post-enrichment (N termini reflecting intact and proteolytic proteoforms) samples analyzed by DDA and label free quantification. *C*, Proteins identified exclusively in plasma before or after N termini enrichment, or in both preparations, mapped to known concentrations in plasma. *D*, t-sne dimensionality reduction of protein N termini abundances followed by fuzzy c-means clustering. Annotation based on spatio-temporal N termini abundance profiles for each cluster as detailed in supplemental Fig. S7*C*. *E*, Relative intensity-based quantification of complement pathway proteins (blue) and N termini identifying previously described activation products (orange).

Finally, we established HUNTER on a basic liquid handling system in 96-well format to enable high-throughput sample analysis and to reduce variation introduced by manual pipetting. Automated enrichment of protein N termini from 1 to 5 μl of human plasma in four technical replicates achieved an improved intra-assay Pearson correlation of 0.93. The interassay Pearson correlation for automated assays on different days and different chromatography columns was 0.86 on average (Fig. 4*A*). We then tested the utility of the automated platform for the characterization of liquid biopsies from cancer patients. We analyzed 3 μl non-depleted blood plasma (BP) and aspirated bone marrow interstitial fluid (BM) from three pediatric B-cell acute lymphoblastic leukemia (B-ALL) patients before and after induction chemotherapy (Fig. 4*B*, supplemental Table S2). This analysis identified 600 N termini of 244 proteins across all patients, with more low-abundance plasma proteins (31) identified after N termini enrichment than in a standard proteome analysis (Fig. 4*C*). Quantitation, t-sne dimensionality reduction and fuzzy c-means clustering of overall protein abundance as determined from aliquots withdrawn before N termini enrichment (supplemental Fig. 8*A*, S8*B*) and N-terminal peptides identified after enrichment (Fig. 4*D*, supplemental Fig. S8*C*) revealed strong treatment-induced changes. Although this has not been studied for bone marrow interstitial fluid before it is well established that proteolytic processes and cleavage fragments in the peripheral blood change in leukemia and during treatment (43–46). In addition to plasma protein processing, caspase cleaved intracellular proteins released from apoptotic cells have been found in leukemic plasma during chemotherapy (47). We found low abundance plasma proteins in our analysis but did not observe such caspase-processed intracellular proteins. This is likely explained by our late sampling on day 29 of treatment. Intracellular proteins associated with cell death have been observed within the first hours of treatment but are likely rapidly cleared from circulation by the glomerulus (47). At treatment day 29, most chemotherapy sensitive cells will already have undergone apoptosis and intracellular fragments will have been cleared. In contrast, identification of plasma protein N termini revealed interesting differences between blood plasma and bone marrow interstitial fluid. Although only few proteins accumulated differentially in blood plasma and bone marrow interstitial fluid proteolytic proteoforms showed marked differences between both compartments, suggesting the presence of distinct protease activities (Fig. 4*D*). Notably, although C3 and C4 protein did not change or changed only moderately, N termini matching complement protein activation sites (48) showed a marked decrease during chemotherapy (Fig. 4*E*, supplemental Fig. S9), in line with the chemotherapy induced complement defects previously reported in ALL (49).

##### Conclusion

In summary, HUNTER is a highly sensitive, universal and scalable protocol for enrichment of protein N termini from crude protein lysates. HUNTER is well suited for automation even on basic liquid handling systems, as it is based on standard magnetic bead and cartridge technology, does not require protein precipitation and avoids phase separations. We have shown successful application in systems as diverse as rat brain and plant leaf tissue, human plasma, sorted peripheral blood cell populations, subcellular fractions enriched for mitochondria and individual *A. thaliana* seedlings. With sensitive identification and reproducible quantification of >1000 protein termini from starting amounts of as little as 10,000 HeLa cells or 2 μg of protein lysate and >5000 termini from 200 μg of protein lysate, HUNTER enables comprehensive analysis of proteolytic processes and protein N-terminal modifications in microscale samples from a wide range of precious limited biological samples and clinical biopsies.

## DATA AVAILABILITY

MS data have been deposited to the ProteomeXchange Consortium(50) (http://www.proteomexchange.org) via the PRIDE (51) (https://www.ebi.ac.uk/pride/archive/) and MassiVE (https://massive.ucsd.edu/) partner repositories with the following accession numbers: PXD012804 for Arabidopsis vpe0 seedlings experiments, PXD012821 for HUNTER termini enrichment with Arabidopsis leaf extracts, PXD012844 for HUNTER termini enrichment with rat brain extracts, PXD012915 for development of HUNTER on HeLa cells and commercially-available plasma; PXD012918 for analysis of sorted human peripheral blood mononuclear cells by HUNTER, PXD012916 for analysis of proteolytic processes in plasma and bone marrow interstitial fluid of B-ALL patients by HUNTER, PXD012919 for analysis of mitochondrial N termini by HUNTER, and PXD014931 for comparison of enrichment performance between HUNTER and TAILS.

## Supplementary Material

supplemental Fig. S7C

"Figures S1-S9 and Tables S1-S2"

Supplementary Tables S3-S12

Supplementary Datasets 1-11

## Footnotes

^1^ The abbreviations used are:

HUNTER: high-efficiency undecanal-based N termini enrichment
AML: acute myeloid leukemia.
