# Supplementary material for: Sensitive Determination of Proteolytic Proteoforms in Limited Microscale Proteome Samples
Source: Mol Cell Proteomics. 2019 Aug 30;18(11):2335–47. doi: 10.1074/mcp.TIR119.001560 (PMC6823850; doi:10.1074/mcp.TIR119.001560)
Supplement: supplemental Fig. S7C [file TIR119.001560_index.html]

Supplement to Sensitive determination of proteolytic proteoforms in limited microscale proteome samples | Molecular & Cellular Proteomics

## Supplemental Data

- "Figures S1-S9 and Tables S1-S2"
- Supplementary Tables S3-S12 - Table S3. Identified N termini on HeLa samples with Data-dependent acquisition (DDA) mode Table S4. Identified N termini on high-pH fractionated HeLa samples with Data-dependent acquisition (DDA) mode Table S5. Identified N termini on HeLa samples with Data-independent acquisition (DIA) mode Table S6. N termini identification from automated post-HUNTER commerically-available plasma samples Table S7. Peptide identification in B-ALL patient plasma (BP) and bone marrow interstitial fluid (BM) pre-HUNTER samples Table S8. N termini identification in B-ALL patient plasma (BP) and bone marrow interstitial fluid (BM) post-HUNTER samples Table S9. N termini identification on peripheral blood mononuclear cells post-HUNTER samples Table S10. List of mitochondrial N terminome from 2.5 million cells by HUNTER Table S11. N termini identification on three VPE0 quadruple mutant seedlings Table S12. N termini identification on single VPE0 quadruple mutant seedlings
- Supplementary Datasets 1-11 - Peptide sequence identifications as reported by MaxQuant
