# Supplementary material for: Sensitive Determination of Proteolytic Proteoforms in Limited Microscale Proteome Samples
Source: Mol Cell Proteomics. 2019 Aug 30;18(11):2335–47. doi: 10.1074/mcp.TIR119.001560 (PMC6823850; doi:10.1074/mcp.TIR119.001560)
Supplement: "Figures S1-S9 and Tables S1-S2" [file 152783_1_unknown_upload_385761_pwwtds.pdf]

Sensitive determination of proteolytic proteoforms in limited microscale proteome samples

Supplementary Material

Samuel S.H. Weng[1,2,5], Fatih Demir[3,5], Enes K. Ergin[1,2], Sabrina Dirnberger[3], Anuli Uzozie[1,2], Domenic Tuscher[3], Lorenz Nierves[1,2], Janice Tsui[1,2], Pitter F. Huesgen[3,4,*] and Philipp F. Lange[1,2,*]

Table S1 | Data matching

Figures	Content	Reference
Fig 1. and S4	HUNTER optimization on HeLa cells with DDA	Table S3 and Dataset 1
Fig 1.	HUNTER optimization on high-pH fractionated HeLa cells with DDA	Table S4 and Dataset 2
Fig 1. and S4	HUNTER optimization on HeLa cells with DIA	Table S5
Fig 2. and S5	N termini identification on peripheral blood mononuclear cells post-HUNTER samples	Table S9 and Dataset 6
Fig 2. and S5	List of mitochondrial N terminome from 2.5 million cells	Table S10 and Dataset 7
Fig 3, S6, and S7	N termini identification on single and three VPE0 quadruple mutant seedlings	Table S11, S12, Dataset 10, and 11
Fig 4.	Termini identification from automated post-HUNTER commercially-available plasma samples	Table S6 and Dataset 3
Fig 4., S8, and S9	N termini identification in B-ALL patient plasma (BP) and bone marrow interstitial fluid (BM) pre-HUNTER samples	Table S7 and Dataset 4
Fig 4. and S9	N termini identification in B-ALL patient plasma (BP) and bone marrow interstitial fluid (BM) post-HUNTER samples	Table S8 and Dataset 5
Fig S3.	Comparison of enrichment performance between HUNTER and TAILS	Dataset 8 and 9

Supplementary Figures

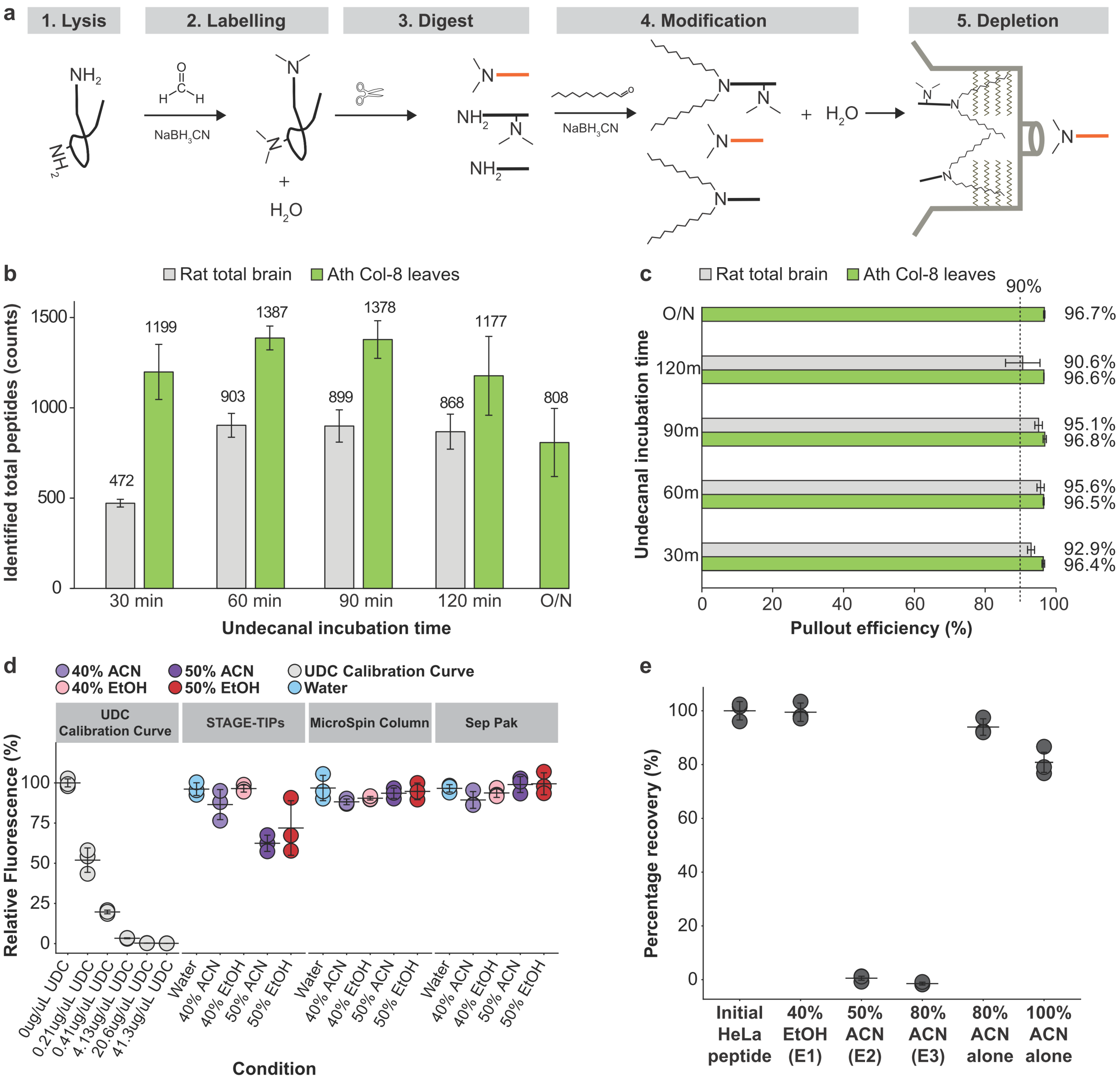

Fig. S1 | Workflow optimization I – Hydrophobic alkylation reaction time and C18 removal. (**a**) Schematic diagram of chemical reactions in HUNTER workflow. (**b,c**) Effect of incubation time for undecanal modification of peptides on the number of identified N-terminal peptides (**b**) and pullout efficiency (**c**). 200µg of rat total brain and *Arabidopsis* Col 8 whole leaf lysates are used as starting material. Mean of n=3 biological replica, error bars indicate SD. (**d**) Effect of different C18 cartridges and mobile phases (EtOH and ACN) on the depletion of free undecanal. Eluent is incubated with tryptic peptides followed and measured using an amine reactive quantitative fluorometric peptide assay to determine the relative amount of free primary amines on tryptic peptides after reaction with undecanal from the eluent. The UDC calibration curve uses free undecanal. n=3 technical replicates, error bars indicate SD. (**e**) Evaluation of peptide recovery from C18 stage-tips using 40% EtOH as mobile phase. Recovery measured by the amine-reactive colorimetric peptide assay relative to the starting material. In a sequential elution using 40% EtOH followed by 50% ACN and 80%. Tryptic peptides de-salted on C18 using 60% ACN were used as starting material. Mean of n=3 technical replicates, error bars indicate SD.

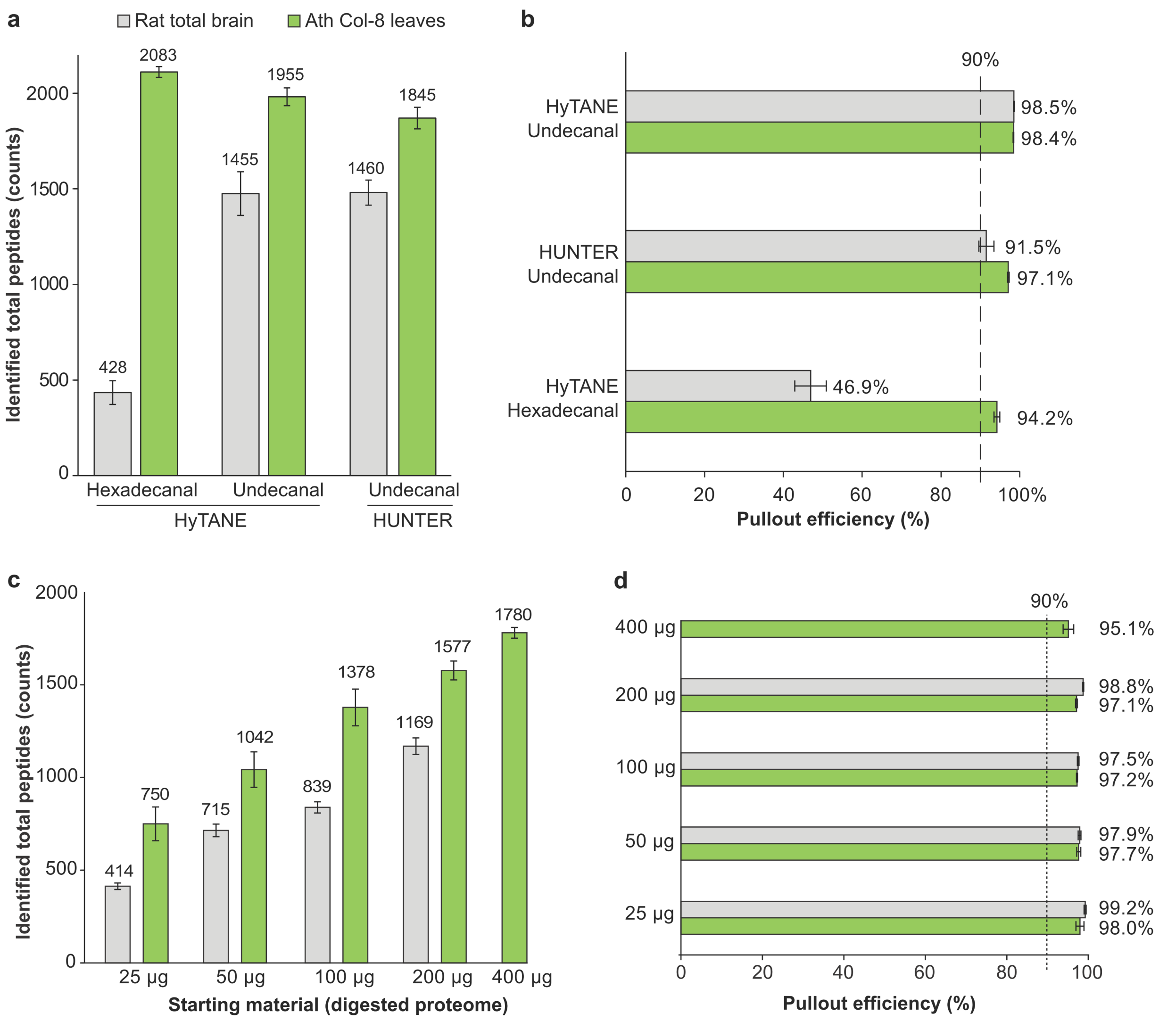

Fig. S2 | Workflow optimization II – Fatty aldehyde chain length and input amount. (**a**) Peptides identified with adapted hexadecanal or undecanal-based HyTANE protocols with removal of organic solvent after tagging before C18-mediated depletion and after direct depletion of tagged peptides in the presence of organic solvent as implemented in HUNTER. (**b**) Enrichment efficiency assessed as N-terminally modified peptides compared to number of digest-generated peptides with free α-amine. (**c, d**) Number of peptides and fraction of N-terminal peptides identified from different amounts of tryptic peptides used for undecanal modification. t=90 min undecanal modification. Mean of n=3 biological replicates, error bars indicate SD. A maximum of 1 µg enriched peptides was analysed by nano-LC-MS/MS.

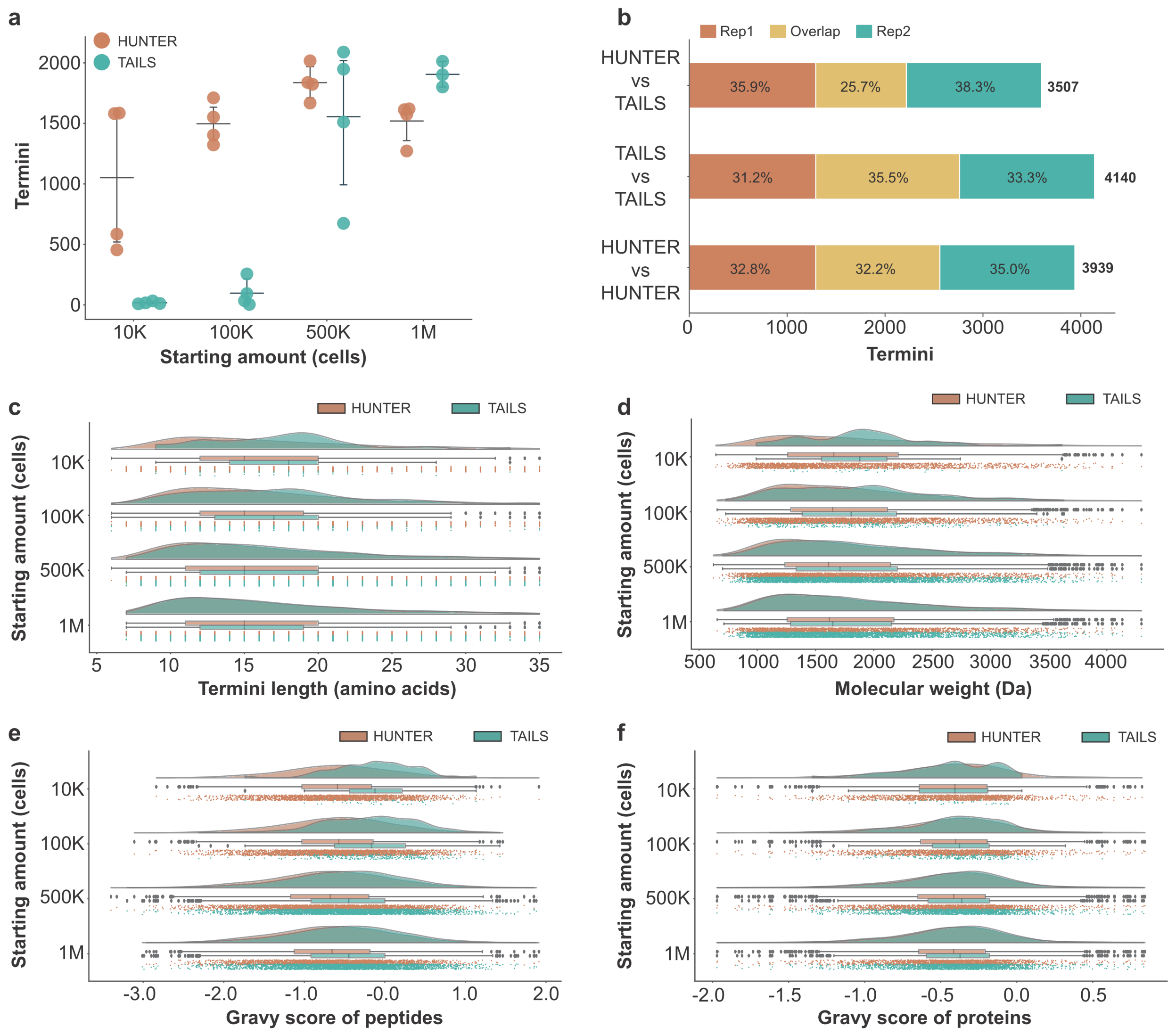

Fig. S3 | Comparison of enrichment performance between HUNTER and TAILS. (**a**) Number of N termini identified from 10,000 to 1,000,000 HeLa cells with HUNTER (orange) and TAILS (green) enrichment methods. Datapoints represent technical replicates (n=4 for each condition, except n=3 for 1M HeLa cells from TAILS), middle lines indicate the mean, and error bars indicate SD. (**b**) Comparison of termini identification in 500K HeLa cells duplicates from two different enrichment methods and within the two enrichment methods. First replica in orange color, second replica in green, and overlap in yellow. (**c**) Peptide length distribution in identified termini from 10,000 to 1,000,000 HeLa cells with HUNTER (orange) and TAILS (green). (**d**) Molecular weight distribution in identified termini from 10,000 to 1,000,000 HeLa cells with HUNTER (orange) and TAILS (green). (**e**) Peptide gravy score distribution in identified termini from 10,000 to 1,000,000 HeLa cells with HUNTER (orange) and TAILS (green). (**f**) Protein gravy score distribution in identified proteins from 10,000 to 1,000,000 HeLa cells with HUNTER (orange) and TAILS (green).

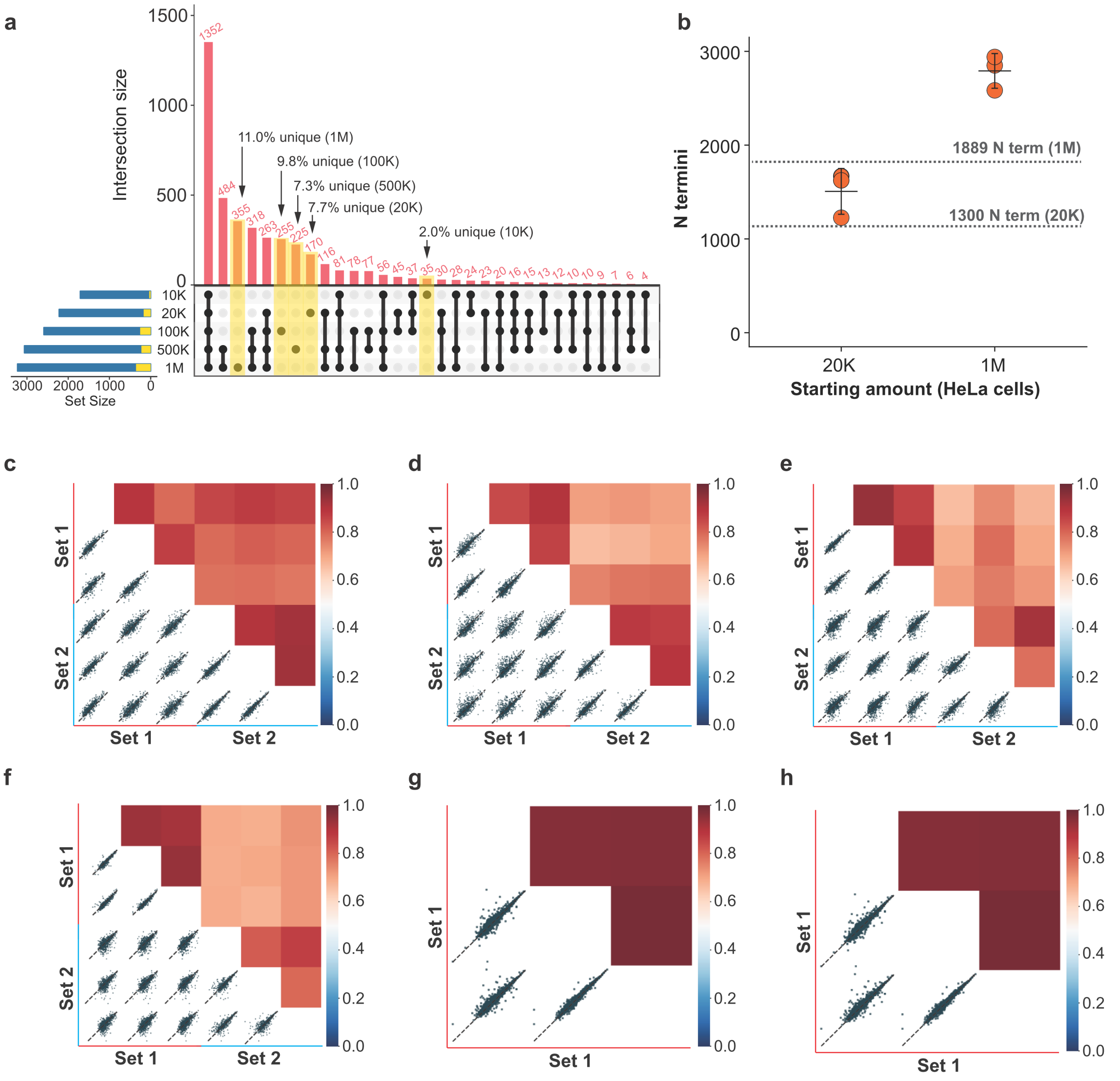

Fig. S4 | Workflow optimization III – Reproducibility. (**a**) Set relationships between different starting amount from 10K to 1M HUNTER-enriched HeLa cells. Percent unique termini are highlighted on top of the associated bars and also highlighted in yellow in the blue set size bars. (**b**) Number of N termini identified from 20K and 1M HeLa cells using DIA. Spectral library built on DDA analysis of 12 high-pH fractions from 1M HUNTER-enriched HeLa cells. Dashed lines show the average number of N termini identified from 20K or 1M HeLa cells by DDA. Mean of n=3 technical replicates. (**c**-**f**) Pearson-correlation between two manually handled HUNTER sets on 10K (**c**), 100K (**d**), 500K (**e**) and 1M (**f**) HeLa cells by DDA. The correlation is based on peptide intensity, n=3 technical replicates. (**g**,**h**) Pearson-correlation between three manually handled HUNTER replicates from 20k (**g**) and 1M (**h**) HeLa quantified by DIA. The correlation is based on sum of elution groups (precursors).

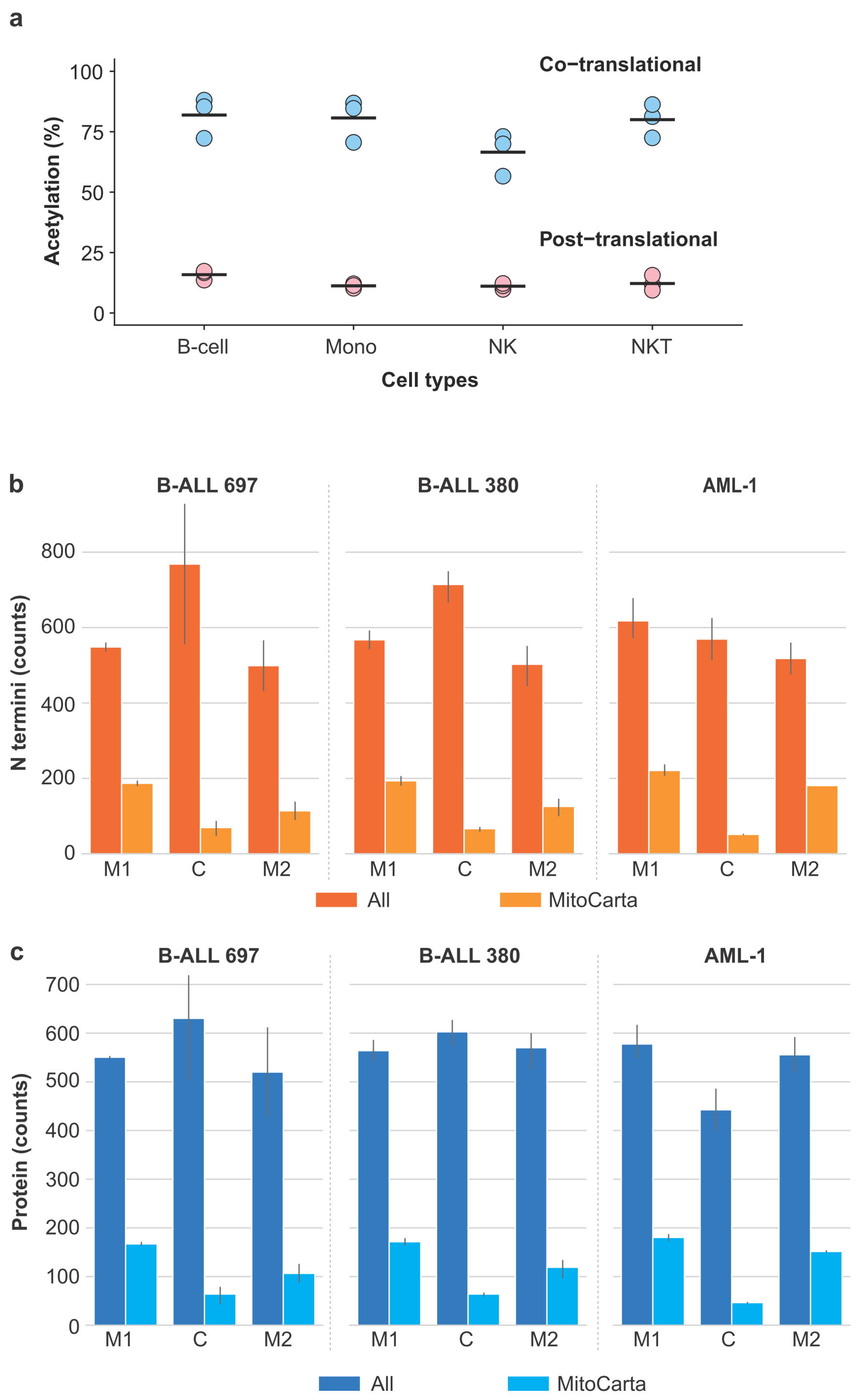

Fig. S5 | Analysis of sorted PBMC and Mitochondrial N terminomes by HUNTER. (**a**) Number of N termini with co-translational (genome encoded position 1 or 2) or post-translational (position >2) acetylation. No significant differences (t test) between cell types were observed. (**b**) N termini identified in each fraction obtained by mild PCT assisted lysis of 2.5 million cells followed by differential centrifugation. N termini annotated as mitochondrial by the MitoCarta resource are displayed in light orange. n=2-3, error=SD. (**c**) The total number of proteins identified by N termini in each fraction. Proteins annotated as mitochondrial by the MitoCarta resource are displayed in light blue. n=2-3, error=SD.

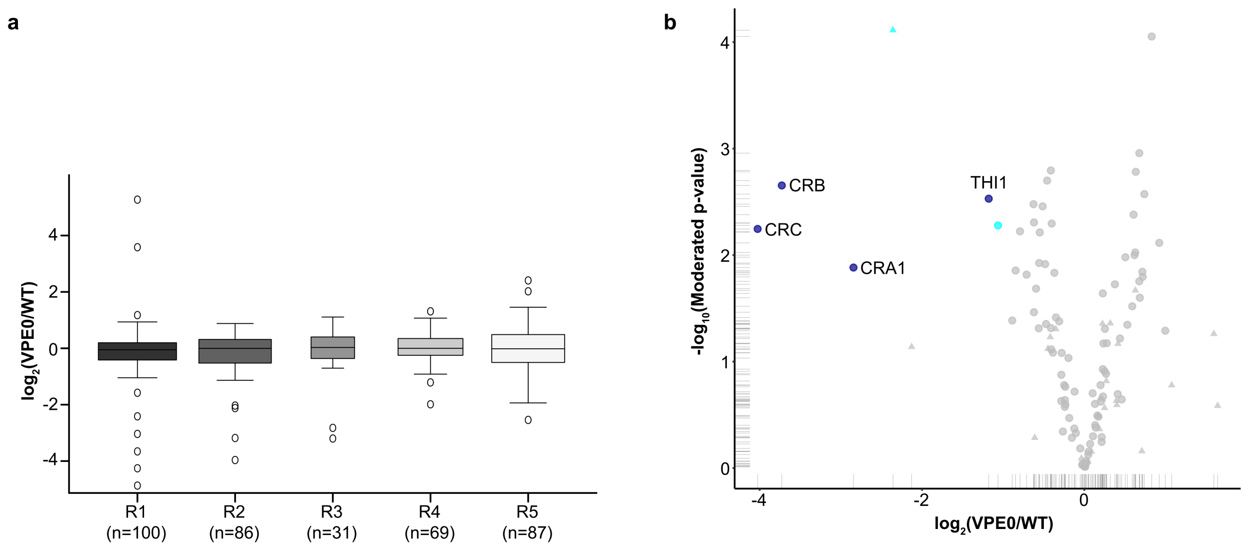

Fig. S6 | Identification and quantification of protein N termini in single *Arabidopsis* seedlings. (**a**) distribution of $\log_2$ ratios in VPE0/wt *Arabidopsis* single seedling HUNTER experiments. (**b**) N termini alterations in 5d old *Arabidopsis*, single VPE0 quadruple mutant/WT seedlings: mainly 12S seed storage proteins (CRA1, CRB, CRC) and THI1 (Thiamine thiazole synthase, chloroplastic) are alternatively processed.

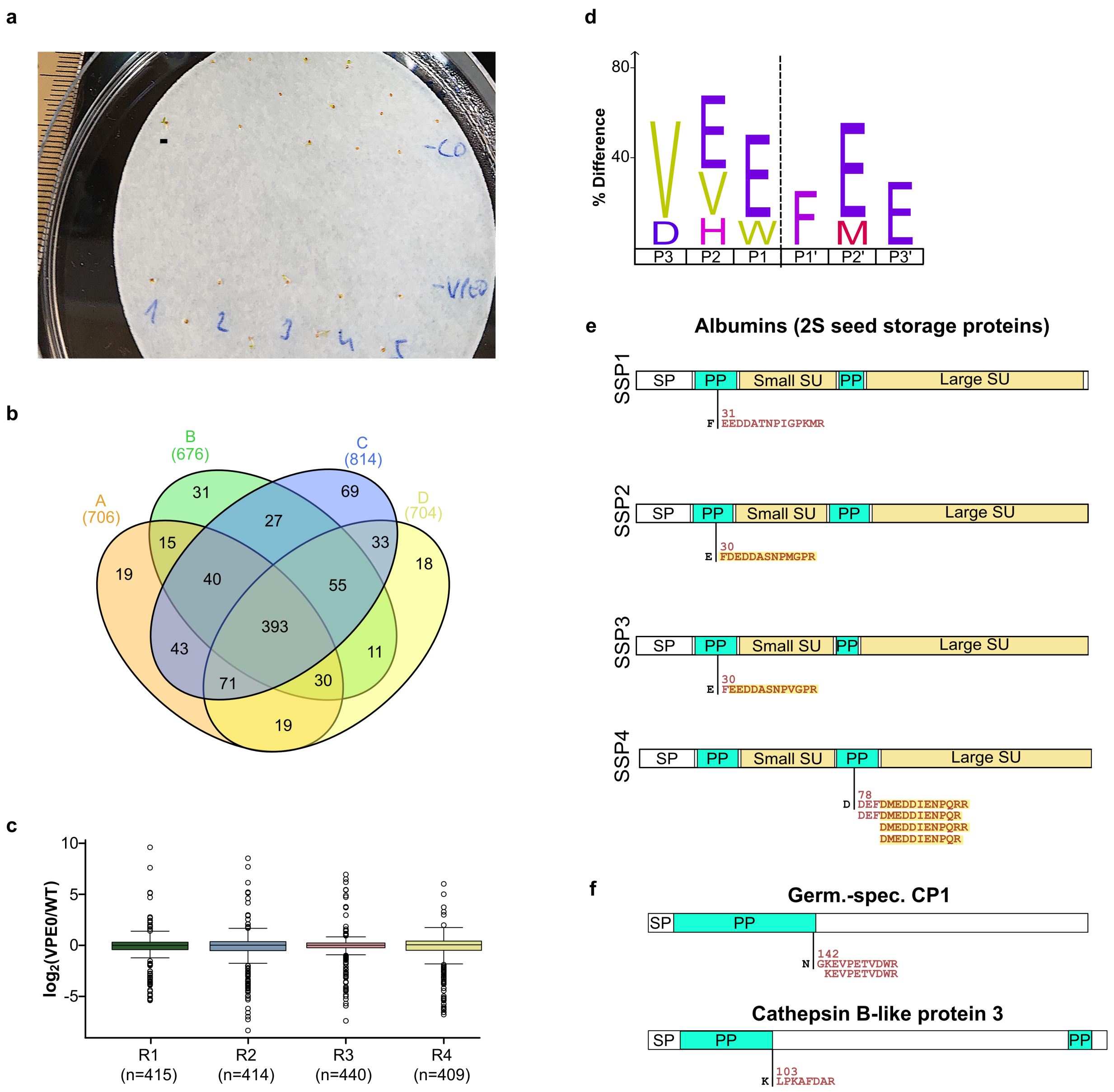

Fig. S7 | Identification and quantification of protein N termini in *Arabidopsis* seedlings. (**a**) Picture of 2.5d old *Arabidopsis* Col-8 wildtype control (CO) and VPE0 quadruple mutant (VPE0) seedlings, grown on filter paper. Scale bar = 1 mm. (**b**) Venn diagram of protein N termini identified by HUNTER with proteomes extracted from three VPE0 and three Col-8 seedlings per experiment, n=4 biological replicates (12 seedlings for each line in total). (**c**) distribution of $\log_2$ ratios in VPE0/wt). (**d**) iceLogo representing 7 filtered cleavage sites deduced from 10 unexpected N-terminal peptides significantly more abundant in the VPE0 mutant. (**e**) proteolytic processing sites in 2S seed storage proteins significantly enriched in WT seedlings. Yellow shaded peptides have already been identified previously (PP: propeptide, SU: subunit). (**f**) Two examples of significantly enriched proteolytic processing sites indicating pro-peptide removal and activation in two proteases.

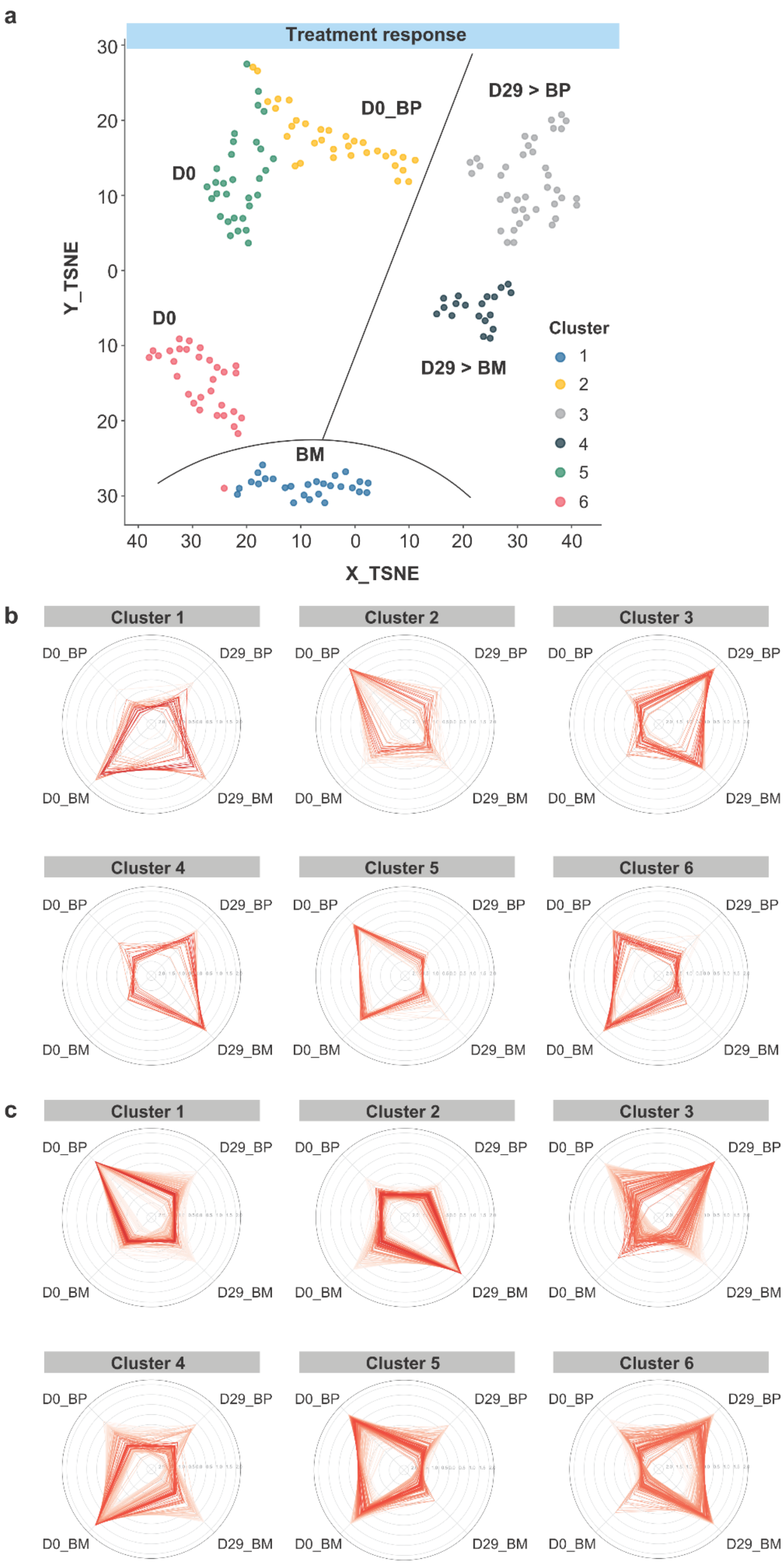

Fig. S8 | Automated HUNTER analysis of blood plasma (BP) and bone marrow interstitial fluid (BM) from three pediatric B-ALL patients at diagnosis (D0) and after induction chemotherapy (D29). (**a**) Unsupervised t-distributed stochastic neighbor embedding (t-SNE) plot for individual quantified proteins split into 6 clusters by fuzzy c-means. Annotation based on spatio-temporal protein abundance profiles. (**b**) Radar plot representation the spatio-temporal abundance profiles of proteins assigned to the 6 clusters identified from (a). (**c**) Radar plot representation for the spatio-temporal abundance profiles of proteins assigned to the 6 clusters identified from (Fig. 4d). Radial axis represents protein or N termini z-score. Color scaled by fuzzy c-means cluster membership score (light to dark red indicating increased cluster membership fit).

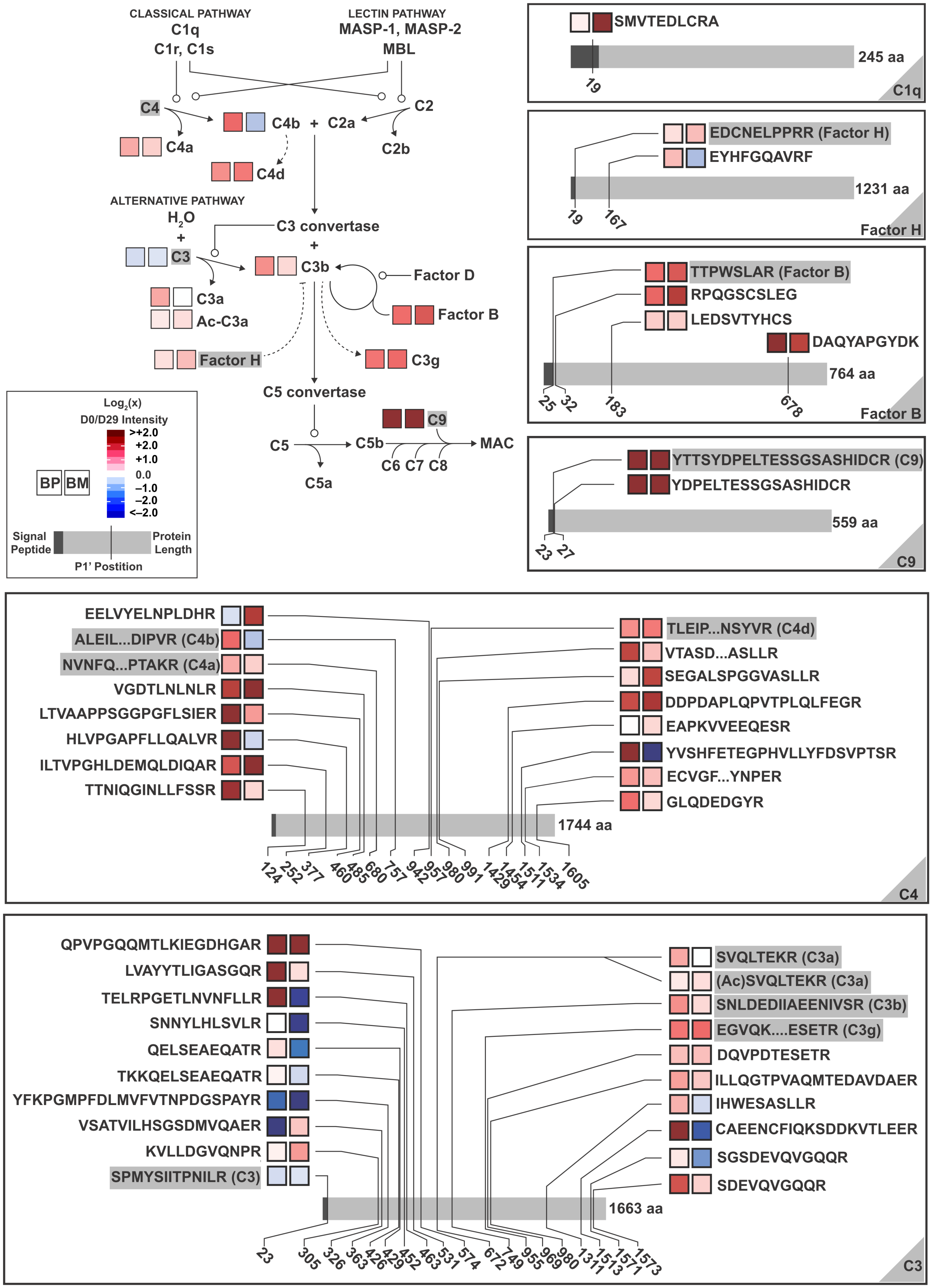

CLASSICAL PATHWAY
C1q
C1r, C1s
LECTIN PATHWAY
MASP-1, MASP-2
MBL
C4
C4b + C2a
C2
C4a
C2b
C4d
ALTERNATIVE PATHWAY
H_2O
+
C3
C3 convertase
+
C3b
Factor D
C3a
Ac-C3a
Factor B
Factor H
C3g
C5 convertase
C9
C5
C5b
MAC
C6 C7 C8
C5a
$Log_2(x)$
D0/D29 Intensity
>+2.0
+2.0
+1.0
0.0
−1.0
−2.0
<−2.0
BP
BM
Signal Peptide
Protein Length
P1' Postition
SMVTEDLCRA
245 aa
19
C1q
EDCNELPPRR (Factor H)
EYHFGQAVRF
1231 aa
19
167
Factor H
TTPWSLAR (Factor B)
RPQGSCSLEG
LEDSVTYHCS
DAQYAPGYDK
764 aa
25
32
183
678
Factor B
YTTSYDPELTESSGSASHIDCR (C9)
YDPELTESSGSASHIDCR
559 aa
23
27
C9
EELVYELNPLDHR
ALEIL...DIPVR (C4b)
NVNFQ...PTAKR (C4a)
VGDTLNLNLR
LTVAAPPSGGPGFLSIER
HLVPGAPFLLQALVR
ILTVPGHLDEMQLDIQAR
TTNIQGINLLFSSR
TLEIP...NSYVR (C4d)
VTASD...ASLLR
SEGALSPGGVASLLR
DDPDAPLQPVTPLQLFEGR
EAPKVVEEQESR
YVSHFETEGPHVLLYFDSVPTSR
ECVGF...YNPER
GLQDEDGYR
1744 aa
124 252 377 460 485 680 757 942 957 980 991 1429 1454 1511 1534 1605
C4
QPVPGGQMTLKIEGDHGAR
LVAYYTLIGASGQR
TELRPGETLNVNFLLR
SNNYLHLSVLR
QELSEAEQATR
TKKQELSEAEQATR
YFKPGMPFDLMVFVTNPDGSPAYR
VSATVILHSGSDMVQAER
KVLLDGVQNPR
SPMYSIITPNILR (C3)
SVQLTEKR (C3a)
(Ac)SVQLTEKR (C3a)
SNLDEDIIAEENIVSR (C3b)
EGVQK....ESETR (C3g)
DQVPDTESETR
ILLQGTPVAQMTEDAVDAER
IHWESASLLR
CAEENCFIQKSDDKVTLEER
SGSDEVQVGQQR
SDEVQVGQQR
1663 aa
23 305 326 363 426 429 452 463 531 574 672 749 955 969 980 1311 1513 1571 1573
C3

Fig. S9 | Regulation of the complement pathway in blood plasma (BP) and bone marrow interstitial fluid (BM) of three pediatric B-ALL patients at diagnosis (D0) and after induction chemotherapy (D29). Termini are displayed according to their position on the genome encoded sequence of proteins involved in the complement pathway. Complement proteins are known to be proteolytically cleaved and several fragments with defined start sites are known. Termini matching these start sites are highlighted by grey background and also added to the pathway diagram. Log_2 fold changes of termini abundance at diagnosis (D0) and after induction chemotherapy (D29) are color coded from blue (<-2) to red (>2) and visualized for blood plasma (BP) and bone marrow interstitial fluid (BM) next to the respective terminus identified by HUNTER**.**

Table. S2 | B-ALL patient clinical data.

Samples	Gender	Ages	WBC Count (D0; $*10^9$/L)	WBC Count (D29; $*10^9$/L)	Peripheral blast count (D0; $*10^9$/L)	Blast % in BM (D0)	MRD (D29 by flow)	Cytogenetics	Immunophenotype
**B-ALL-1**	M	4y8m	6.4	5.1	1.15	73%	1.05%	Translocation (12;21) with fusion ETV6/ RUNX1; complex karyotype; clonal evolution	The marrow sample contains a dominant population of cells expressing very dim CD45. This population expresses B cell markers CD19, cCD79a and cCD22. Overall the findings are consistent with B-ALL, with aberrant expression of CD15. e.g dim CD45, CD19+, cCD79a+, cCD22+
**B-ALL-2**	M	12y9m	1.2	6.5	None	79%	<0.01%	Fusion ETV6/ RUNX1, normal male karyotype	The marrow sample contains a dominant population of cells comprising 89% of events and expressing very dim CD45 vs low side scatter. This population expresses CD10, CD19, cCD79a, cCD22, partial CD34, CD38, TdT, and HLA-DR. Overall the findings are consistent with B-ALL, with possible aberrant expression of myeloid markers CD13 and CD33.
**B-ALL-3**	M	4y8m	2.9	5.8	0.09	93%	<0.01%	A hyperdiploid clone with 58-60 chromosomes. Marrow specimen has a high hyperdiploid clone with segmental loss of CDKN2A.	The marrow sample contains a dominant population of cells comprising 88% of all events and expressing very dim CD45 vs low side scatter. This population expresses CD10, CD19, dim partial CD20, cCD79a, cCD22, cMu, CD34, TdT, and HLA-DR. Overall the findings are consistent with B-ALL.

Note: white blood cell abbreviated as WBC; bone marrow abbreviated as BM; minimal residual disease abbreviated as MRD; D0 is before treatment whereas D29 is after treatment.
